# FRK inhibits breast cancer cell migration and invasion by suppressing epithelial-mesenchymal transition

**DOI:** 10.18632/oncotarget.22958

**Published:** 2017-12-06

**Authors:** Yetunde Ogunbolude, Chenlu Dai, Edward T Bagu, Raghuveera Kumar Goel, Sayem Miah, Joshua MacAusland-Berg, Chi Ying Ng, Rajni Chibbar, Scott Napper, Leda Raptis, Frederick Vizeacoumar, Franco Vizeacoumar, Keith Bonham, Kiven Erique Lukong

**Affiliations:** ^1^ Department of Biochemistry, College of Medicine, University of Saskatchewan, Saskatoon, Canada; ^2^ Cancer Research Unit, Health Research Division, Saskatchewan Cancer Agency, and Division of Oncology, College of Medicine, University of Saskatchewan, Saskatoon, Canada; ^3^ Department of Pathology Royal University Hospital Saskatchewan, Saskatoon, Canada; ^4^ Departments of Pathology & Laboratory Medicine, University of Kansas Medical Center, Kansas City, MO, USA; ^5^ Vaccine and Infectious Disease Organization, University of Saskatchewan, Saskatoon, Canada; ^6^ Departments of Microbiology and Immunology and Pathology, Queen's University, Kingston, Canada

**Keywords:** fyn-related kinase -FRK, breast cancer subtypes, triple negative breast cancer, basal A and B, STAT3

## Abstract

The human fyn-related kinase (FRK) is a non-receptor tyrosine kinase known to have tumor suppressor activity in breast cancer cells. However, its mechanism of action has not been fully characterized. We generated FRK-stable MDA-MB-231 breast cancer cell lines and analyzed the effect on cell proliferation, migration, and invasiveness. We also used kinome analysis to identify potential FRK-regulated signaling pathways. We employed both immunoblotting and RT-PCR to identify/validate FRK-regulated targets (proteins and genes) in these cells. Finally, we interrogated the TCGA and GENT gene expression databases to determine the correlation between the expression of FRK and epithelial/mesenchymal markers. We observed that FRK overexpression suppressed cell proliferation, migration, and invasiveness, inhibited various JAK/STAT, MAPK and Akt signaling pathways, and suppressed the expression of some STAT3 target genes. Also, FRK overexpression increased the expression of epithelial markers including E-cadherin mRNA and down-regulated the transcript levels of vimentin, fibronectin, and slug. Finally, we observed an inverse correlation between FRK expression and mesenchymal markers in a large cohort of breast cancer cells. Our data, therefore, suggests that FRK represses cell proliferation, migration and invasiveness by suppressing epithelial to mesenchymal transition.

## BACKGROUND

A fyn-related kinase (FRK), or protein tyrosine kinase 5 (PTK5), is a non-receptor tyrosine kinase that belongs to the BRK family kinases (BFKs) and is evolutionarily related to Src [1]. The other two members of the family include BRK (Breast tumor kinase) and SRMS (Src-related kinase lacking C-terminal regulatory tyrosine and N-terminal myristoylation sites) [1, 2]. The gene encoding FRK maps to the chromosomal locus 6q22-q23.2, a region frequently deleted owing to loss of heterozygosity (LOH) in nearly 48% of cancers [1]. Like Src family kinases, FRK is functionally composed of a Src homology 3 (SH3) domain, an SH2 domain, a kinase domain and a putative C-terminal regulatory tyrosine (Y497) in addition to a conserved auto-regulatory tyrosine residue (Y387) in its catalytic domain but lacks the N-terminal myristoylation/palmitoylation signals, which increases its ability to interact with intracellular substrates [3]. Also, the FRK SH2 domain contains a bipartite nuclear localization signal, which is known to promote the nuclear localization of FRK [4, 5].

FRK functions have been noted to be regulated in a tissue-specific context. While recent studies have discovered FRK-mediated growth-regulatory properties in the liver and pancreatic cancers, the tumor suppressor properties of FRK have been well studied in breast and glial cancers ([6], [7] and reviewed in [1]). The expression of FRK was detected in breast cancer cell lines such as BT20, MDA-MB-468, and MCF7, but not in BT549, MDA-MB-231, MDA-MB-435 cell lines [4, 8]. Similarly, FRK was absent in 16 of 21 of invasive breast carcinomas studied but detected in normal epithelium [9]. Several studies have reported various potential mechanisms by which FRK acts as a tumor suppressor in glioma and breast cancer cells. For instance, FRK inhibits cell proliferation, migration and invasion in glioma cells by promoting N-cadherin/β-catenin complex formation [10], inhibiting cyclin D1 nuclear accumulation [11] and regulating JNK/c-Jun signaling [12]. However, in breast cancer cells, FRK suppresses cell proliferation by arresting cells in the G1 phase of the cell cycle [5, 13]; regulating PTEN protein stability and function [8]; inhibiting EGFR signaling [14], and by regulating the stability and potentially the tumor suppressor function of BRCA1 protein [15]. Little is known about the role and the mechanism of action of FRK in cell migration and invasion in breast cancer.

Breast cancer is the most frequent female malignancy in the western world and one of the leading causes of cancer-related deaths in women [16]. Breast cancer is a complex and heterogeneous disease that can be classified into at least four molecular subtypes: HER2 (human epidermal growth factor receptor 2), basal, luminal A and luminal B [17–19]: Based on transcriptional analyses of several breast cancer cell lines, Neve and his colleagues further classified breast cancers cell lines into 2 major clusters. These are luminal and basal-like clusters. Basal-like clusters are further divided into basal A and basal B [20]. There is no distinct HER2 cluster. However, HER2 was shown to its among luminal and basal A clusters. These clusters were shown to have distinct morphological and invasive properties. Luminal clusters appear more differentiated, form tight cell-cell junctions and have a noninvasive phenotype [20] while basal B cells appear less differentiated and highly invasive with more mesenchymal-like appearance. However, basal A cells have either luminal-like or basal-like morphologies [20].

The majority of breast cancer-related mortalities result from the migration of *in-situ* breast tumor cells to distal organs such as the lungs, liver, bone, and brain [21]. For such migration to occur, these in-situ breast tumor cells undergo a morphological change from a noninvasive phenotype to a highly invasive, mesenchymal-like phenotype. This is regulated by a process termed Epithelial-to-mesenchymal transition (EMT). EMT is the hallmark characteristic of certain transformed cells that promote the metastatic/invasive potential of these cells [22–24]. Loss of adherens junction proteins, typically E-cadherin, and upregulation of mesenchymal markers such as fibronectin, vimentin, and N-cadherin are major molecular events that dr ive EMT in various cancer cells [22, 23, 25]. A number of reports have shown that tyrosine kinases promote cell invasion and migration by EMT [26, 27].

FRK has been shown to regulate cell proliferation of breast cancer and glioma cells, but its role in cell invasion in breast cancer has not been fully explored. It is also unclear whether the expression of FRK correlates with any breast cancer clinical parameter. In the present study, we found that FRK expression was typically low in the basal B breast cancer cells that exhibit mesenchymal characteristics and provide evidence that FRK regulates EMT in breast cancer cells.

## RESULTS

### FRK expression is high in epithelial-like breast cancer cells and the normal breast epithelium

**Figure 1:**
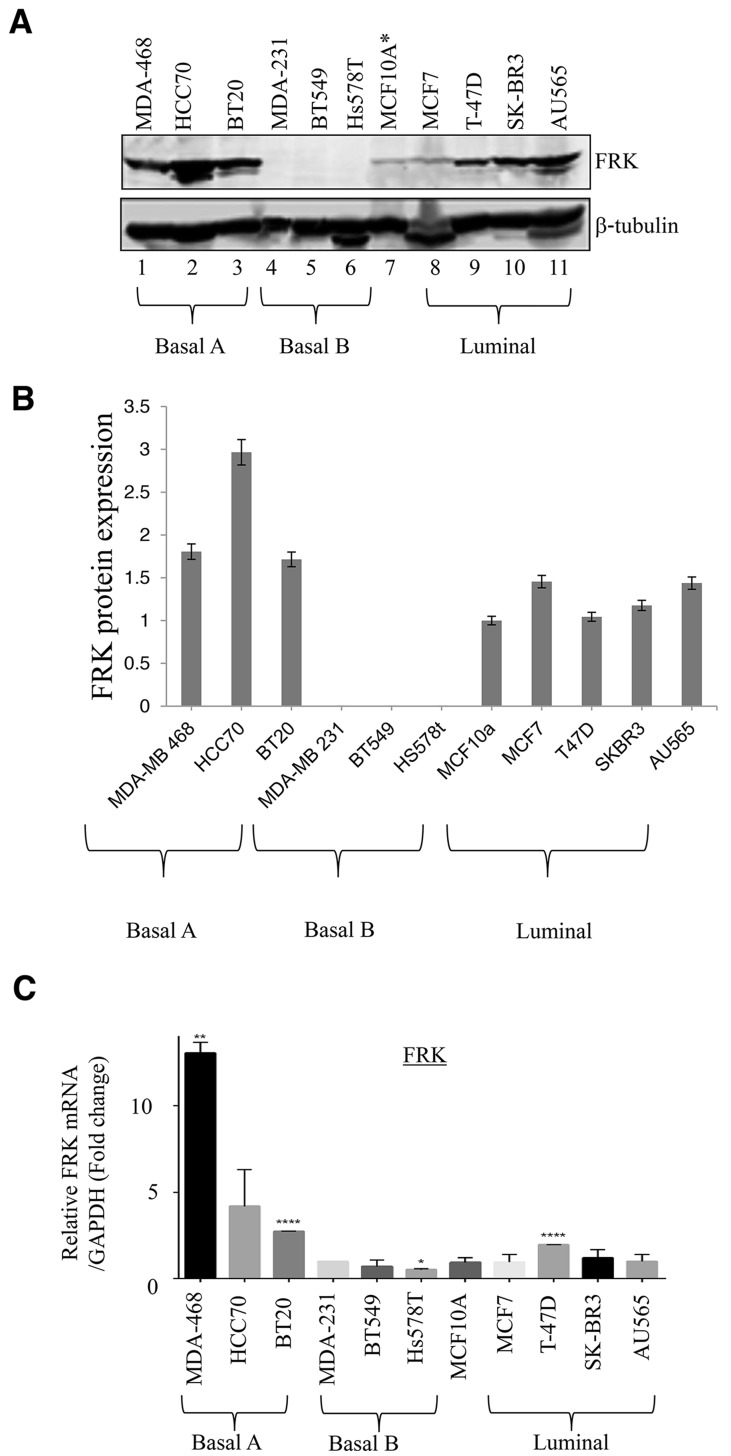
FRK expression in breast cancer cell lines **(A)** The immortalized normal mammary epithelial cell line, MCF10A as well as the indicated breast cancer cell lines, corresponding to either the Basal A, Basal B or the luminal subtypes, were probed for FRK expression. β-tubulin was used as the loading control. **(B)** FRK protein expression was quantified using Image J software. Graph is representative image of the protein expression Figure 1A. **(C)** FRK mRNA levels in the same cell lines were quantitatively determined relative to MCF 10A with RT-PCR analyses using appropriate probes.

Although FRK is thought to be a potential tumor suppressor in breast cancer, past studies investigating the tumor suppressive role of FRK were irrespective of breast cancer subtypes [4, 8]. To take a deeper look at the biological relevance of FRK in breast cancer, we analyzed the expression of FRK in a broader panel of 11 breast cancer cell lines classified into three subtypes (luminal, Basal B and Basal A) based on the cell morphology and invasive potential. Luminal cells are more differentiated with epithelial-like phenotype while the Basal B cells are less differentiated and possess more mesenchymal-like appearance; Basal A cells have either luminal-like or basal-like morphologies [20]. The cells used in this study include AU565, SKBR3, MCF-7 and T47D (luminal), MDA-MB-468, BT20, HCC 70 (Basal A) and MDA-MB-231, Hs 578T, BT549 (Basal B) and MCF10A a non-tumorigenic cell line derived from normal mammary epithelium. The cell lines were analyzed for both FRK protein and mRNA expression. As seen in Figure 1A and 1B, Basal A cell lines showed the highest FRK protein expression, compared to the luminal which displayed moderate levels, and Basal B where the expression of FRK was largely undetectable. The expression in MCF10A was low/moderate. These results were consistent with the mRNA expression data showing high and low expression of FRK transcripts in Basal A and Basal B cell lines, respectively (Figure 1C). These data indicate that FRK is differentially expressed in breast cancer cells and that expression of FRK is higher in epithelial-like cell lines, compared with those with mesenchymal characteristics.

**Figure 2:**
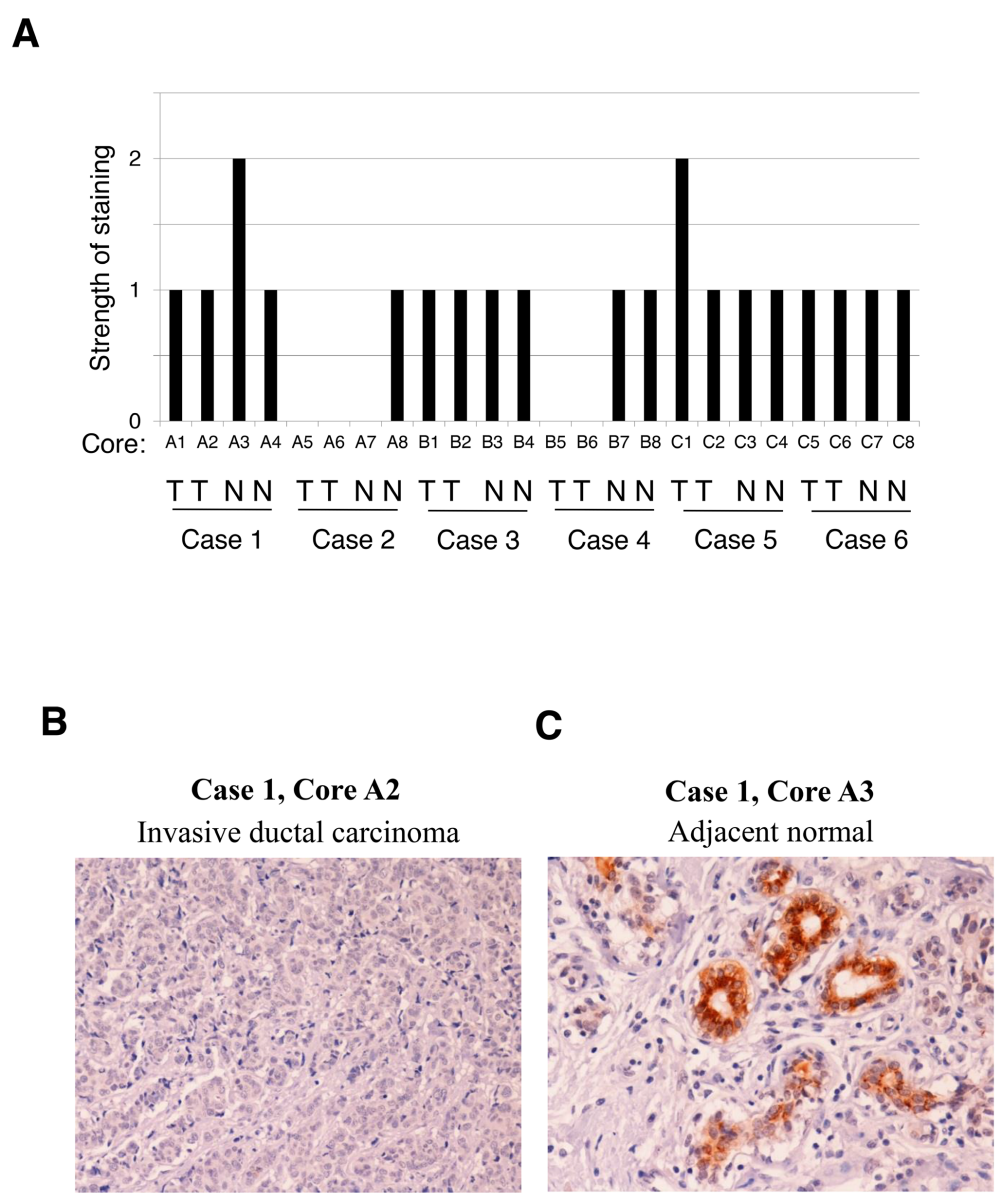
FRK expression in breast cancer tissues FRK expression was determined via immunohistochemistry (IHC) analyses on breast cancer tissue microarray containing 6 cases (24 cores, A1 to C8) of breast invasive ductal carcinoma (T) and matched adjacent normal breast tissue (N). **(A)** FRK staining in each core was scored as negative (0), weak (1), moderate (2), or strong (3). **(B)** and **(C)** Shown here are a representative image of the TMA staining with antibodies against FRK. Case 1, core 2 (Invasive ductal carcinoma) and matched adjacent normal tissue (Case 1, core 3).

Differential FRK mRNA and protein expression between epithelial-like and mesenchymal cells prompted us to investigate FRK protein expression in normal and malignant breast tissue microarray (TMA) samples. The TMA used included TNM, clinical stage and pathology grade, from 6 cases of breast invasive ductal carcinoma and matched adjacent normal breast tissue, with quadruple cores per case (Supplementary Table 2). We performed IHC for FRK expression and scored for staining (negative, 0; weak, 1+; moderate, 2+; or strong, 3+). The scores were then converted to number from 0 to 3 scales and plotted. The total positive cell numbers (summary of weak positive, positive and strong positive numbers) and intensity (Summary of Intensity of Weak Positive, Total Intensity of Positive and Total Intensity of Strong Positive) of anti-FRK staining were computed and measured by TMA Software from Aperio Scanning System (Supplementary Table 3). We found that majority of the samples (22 out of 24) displayed a score of 1 or less and only two samples (one normal and one tumor) showed a 2+ strength (Figure 2A). This indicated that FRK protein expression was low/moderate in both normal and cancer tissues. However, where expressed, FRK was localized predominantly in the epithelial cells of intact mammary ducts in the normal breast tissue (Figure 2B, 2C). No obvious correlation was observed between FRK expression and the clinicopathological characteristics such as tumor grade and TNM in the small sample size used in this study. However, our expression data corresponding to cell lines and TMA together indicate that the expression of FRK is enriched in epithelial cells/tissue and clearly downregulated in mesenchymal-like Basal B breast cancer cells.

### FRK-Y497F mutant is constitutively active compared to wild-type FRK

**Figure 3:**
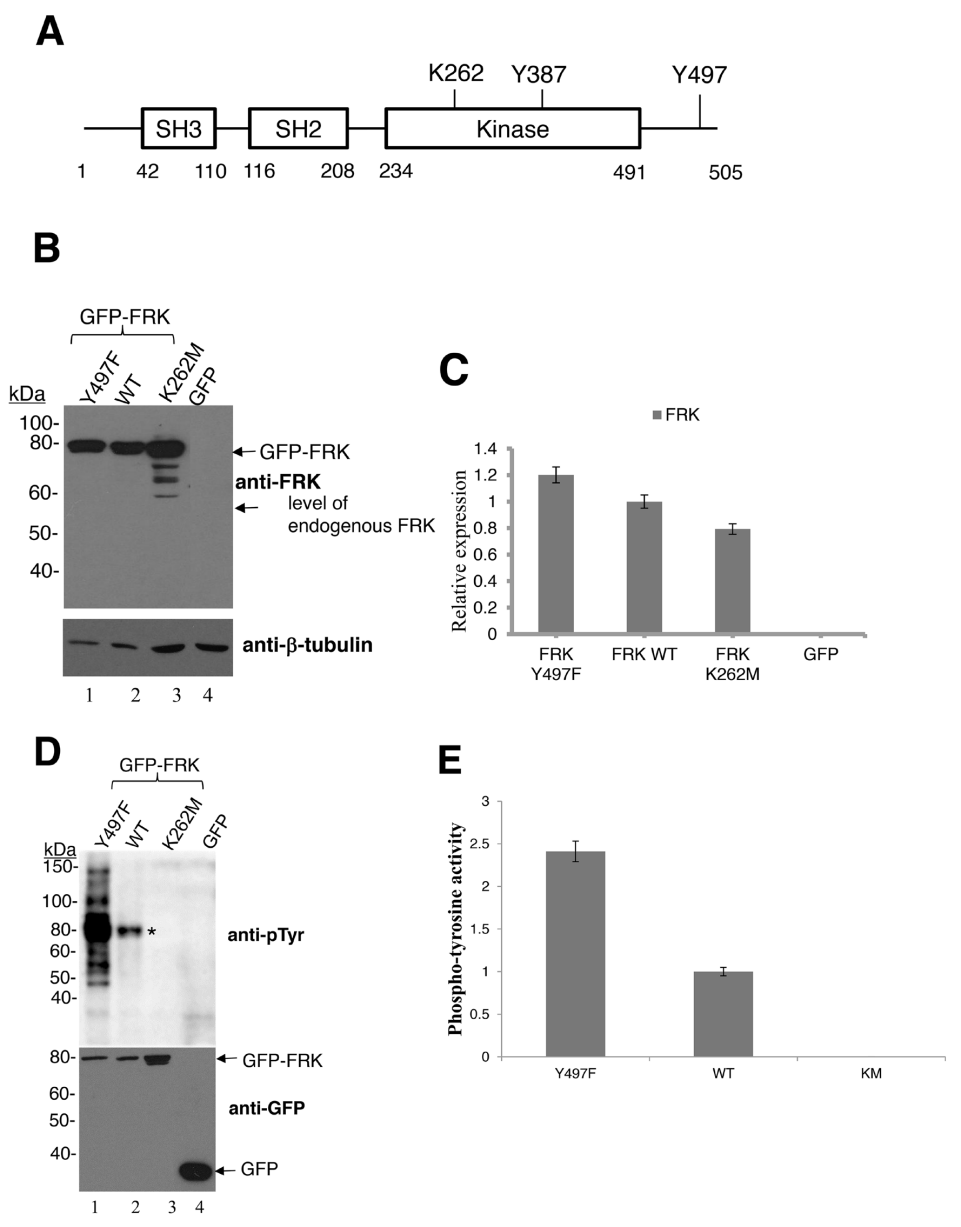
Enzymatic activity of wild-type FRK and FRK variants, FRK K262M and FRK Y497F **(A)** Shown here is a representative figure depicting the FRK functional domains, namely the Src homology 3 (SH3), Src homology 2 (SH2) and the kinase domain. Also shown are the key conserved residues implicated in the regulation of FRK enzymatic activity. These are the ATP-contacting lysine (K262), the autophosphorylation site (Y387) and the C-terminal regulatory tyrosine residue (Y497). **(B)** HEK 293 cells were transiently transfected with either the empty pEGFP-C1 vector or GFP-tagged wild-type FRK (GFP FRK WT), GFP-tagged kinase-dead FRK (GFP-FRK K262R) or a constitutively active FRK variant (GFP-FRK Y497F). Cells were lysed and the lysates resolved via SDS-PAGE and used in Western blotting analyses. Anti-FRK antibody was used to determine the expression of the transfected FRK variants **(C)** Representative image of the blot in figure 3B. Protein expression was quantified using image j software **(D)** Lysates as described in 3B were analyzed for tyrosine kinase activity. Anti-phosphotyrosine antibodies (PY20) were used to determine total tyrosine-phosphorylated proteins in the samples. Anti GFP antibody was used as the loading control. “^*^” indicates the expected position of autophosphorylated FRK. **(E)** Representative image of phosphotyrosine activity shown in Figure 3D, protein expression were quantified using Image J software.

Src-family kinases (SFKs), as well as BRK, are activated by site-specific autophosphorylation on a tyrosine residue and inactivated by phosphorylation of a specific C-terminal tyrosine (Tyr-527 in Src, Tyr-497 in BRK and Tyr-504 in murine FRK) [13, 28, 32]. Mutation of this C-terminal tyrosine has been shown to constitutively activate the enzyme. Human FRK shares the same structural features as these tyrosine kinases and is therefore predicted to have the same mode of enzymatic regulation. The conserved domains include the SH3, the SH2, and the kinase domain. The auto-phosphorylation and ATP binding sites (Y387 and K262, respectively) of FRK are located on the kinase domain, while the Y497 residue lies in the C-terminal region extending from the kinase domain (Figure 3A). The specific enzymes that phosphorylate or dephosphorylate the C-terminal tyrosine of FRK are unknown. To investigate the cellular effect of human FRK activation, we first generated and assayed the activity of various GFP-tagged FRK variants. These variants included FRK-Y497F (activating), FRK K262M (disruption of ATP binding; kinase-defective) and FRK wild-type (WT). These constructs were transiently transfected into HEK 293 cells, which do not express FRK endogenously (Figure 3B). The expression levels of the transiently transfected FRK were comparable (Figure 3B and 3C). Although we observed smaller products produced by FRK-KM mutants, it is possible that the inactive FRK was being degraded. The level of tyrosine phosphorylation (a measure or indication of kinase activity) of the cellular proteins, following transfection with the constructs, were detected by immunoblotting with anti- phosphotyrosine antibody (pY20). Transfection with GFP-FRK-Y497F led to more enhanced phosphorylation of several cellular targets (Figure 3D), compared to FRK-WT (lane 2). Further, strong autophosphorylation of both FRK-Y497F and WT was also observed (indicated by asterisks, Figure 3D). The enhanced kinase activity induced by GFP-FRK-Y497F indicated that the C-terminal tyrosine 497 is essential in the regulation of FRK activity. As predicted, transfection with GFP-FRK-K262M (the catalytically inactive variant) did not result in the phosphorylation of any targets above the control level (Figure 3D lanes 3 and 4; Figure 3E). However, other mutations of FRK kinase domain increased FRK activity in breast cancer cells (Supplementary Figure 1), as previously shown in hepatocellular adenomas [6]. Our result indicates that FRK-Y497F is constitutively active. This mutant can, therefore, serve as a tool to study the cellular effects of fully activated FRK.

### FRK suppresses proliferation in breast cancer cells

**Figure 4:**
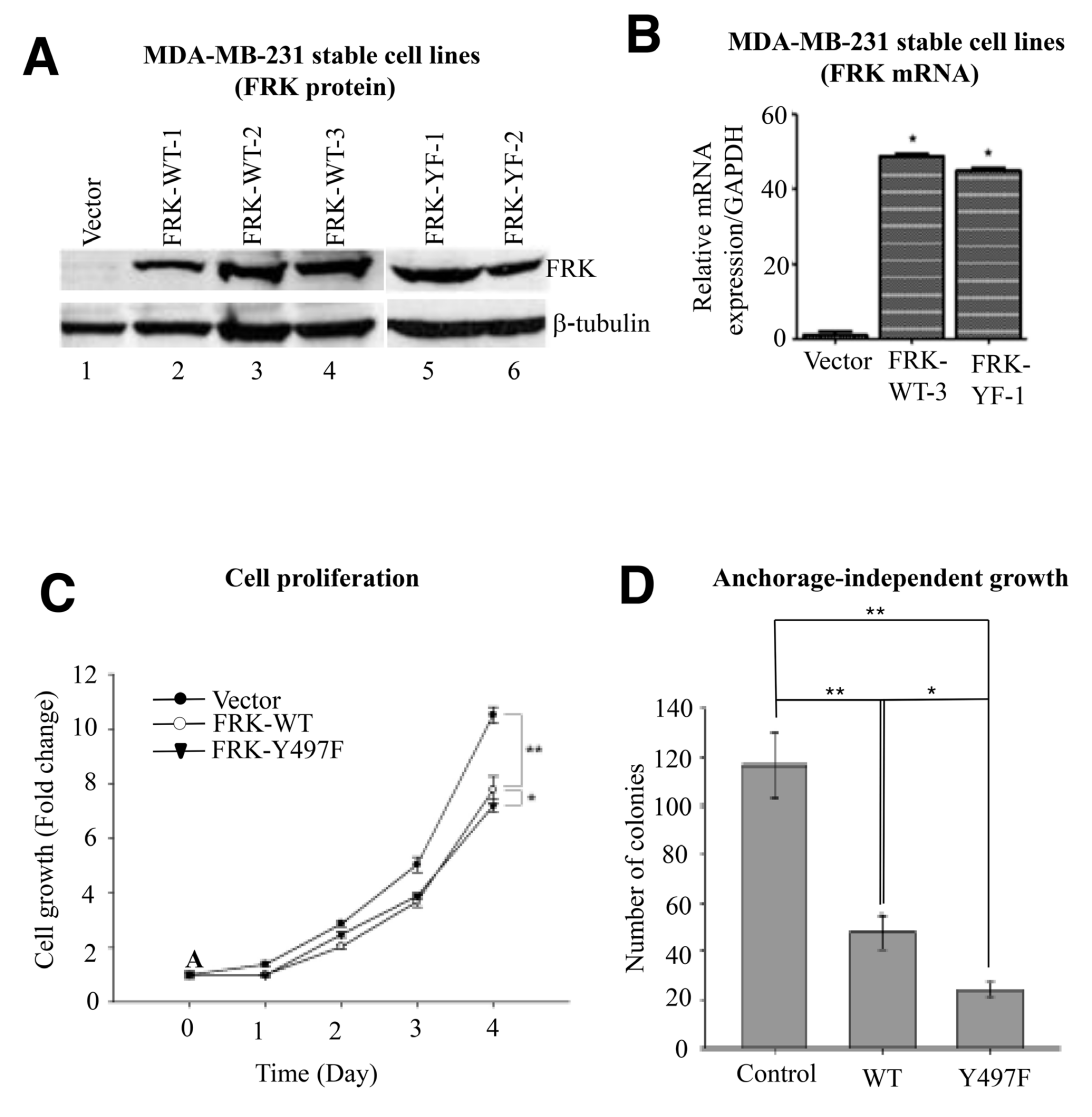
Effect of FRK expression on cell proliferation and anchorage-independent growth **(A)** Vectors encoding wild-type FRK (FRK-WT) or FRK Y497F (FRK-YF) were retrovirally introduced into MDA-MB-231 cells and monoclonal populations derived and designated as indicated. The empty vector-transduced cell line served as control. FRK expression was determined in the stable cell lines by Western blotting analysis using antibodies against FRK. Beta-tubulin served as the loading control. **(B)** FRK mRNA expression in the indicated stable cell lines was determined via quantitative RT-PCR analyses. **(C)** Cell proliferation rates of the indicated FRK stable cell lines and the control cells expressing the empty vector were measured using the MTT assay. **(D)** Soft-agar assays were performed to examine anchorage-dependent growth in the control and FRK-expressing stable cell lines, FRK-WT and FRK Y497F. Statistical analyses for the replicates (n=3) of cell proliferation and soft-agar assays were performed using Graph-pad PRISM software (Ver. 5.0).

FRK wild-type was reported to have growth inhibitory effects on MCF-7 and BT474 breast cancer cells [8]. Given the dramatic effect of constitutively active FRK-Y497F on cellular targets (Figure 3B), we compared the effect of FRK-WT and FRK-Y497F on breast cancer cell proliferation. We generated MDA-MB-231 cell lines stably expressing FRK-WT and FRK-Y497F (See Materials and Methods). We chose MDA-MB-231 because, as shown in Figure 1A and 1C, this cell line expresses significantly lower levels of both FRK mRNA and protein. Further, MDA-MB-231 is a highly metastatic, tumorigenic cell line, and as such is an ideal model system for gain-of-function studies to investigate the effect of FRK on cell proliferation, migration, and invasion. Three FRK wild-type (WT1, WT2, and WT3) and two FRK-Y497F (YF1 and YF2) monoclonal populations, expressing high levels of FRK protein compared with parental MDA-MB-231 cells (control), were selected (Figure 4A). WT3 and YF1 were used in subsequent experiments and referred to as WT, YF or Y497F.The overexpression of FRK in the stable cell lines was also confirmed via RT-PCR analysis (Figure 4B).

To evaluate the effect of FRK on cell proliferation, we employed the Cell Counting Kit-8 (CCK-8) in MDA-MB-231 stably expressing FRK-WT and FRK-Y497F as well as in the parental cells. CCK-8 assay measures dehydrogenase activity in functional mitochondria, which is a direct reflection of the cell viability. MDA-MB-231 cells stably expressing FRK-WT and FRK-Y497F displayed significantly diminished cell proliferation compared with parental cells after 4 days (P < 0.005) (Figure 4C). Similar results were obtained using MDA-MB-231 polyclonal selected cells that stably express FRK-WT and FRK-Y497F. FRK-WT and FRK-Y497F significantly diminished cell proliferation when compared with the parental cells (Supplementary Figure 2). However, we observed no significant effect of FRK kinase-defective mutant (FRK-KM) on cell proliferation and also on cell death when analyzed with 7AAD assays using a flow cytometer when transiently transfected into MDA-MB -231 breast cells (Supplementary Figure 2B, 2C, 2D, 2E and 2F).

We next tested the effect of FRK on the colony-formation ability of MDA-MB-231 cells. There were approximately 120 colonies in the control group, about 50 in the FRK-WT group and on average about 25 in the FRK-Y497 group (Figure 4D). It is interesting to note that the colony sizes of parental MDA-MB-231 cells were greater than that of both the MDA-MB-231-FRK-WT and MDA-MB-231-FRK-Y497F cells (data not shown). The colony numbers in the cells expressing FRK were notably reduced compared with the control group. FRK-Y497F cell number was 50% lower than the FRK-WT numbers (P < 0.005; Figure 4D). Therefore, the effect of activated FRK in inhibiting colony-formation was significantly higher compared with wild-type FRK. Taken together, these results strongly suggest that the activation of FRK contributed to the suppression of cell proliferation and colony formation.

### FRK inhibited breast cancer cell migration and invasion

**Figure 5:**
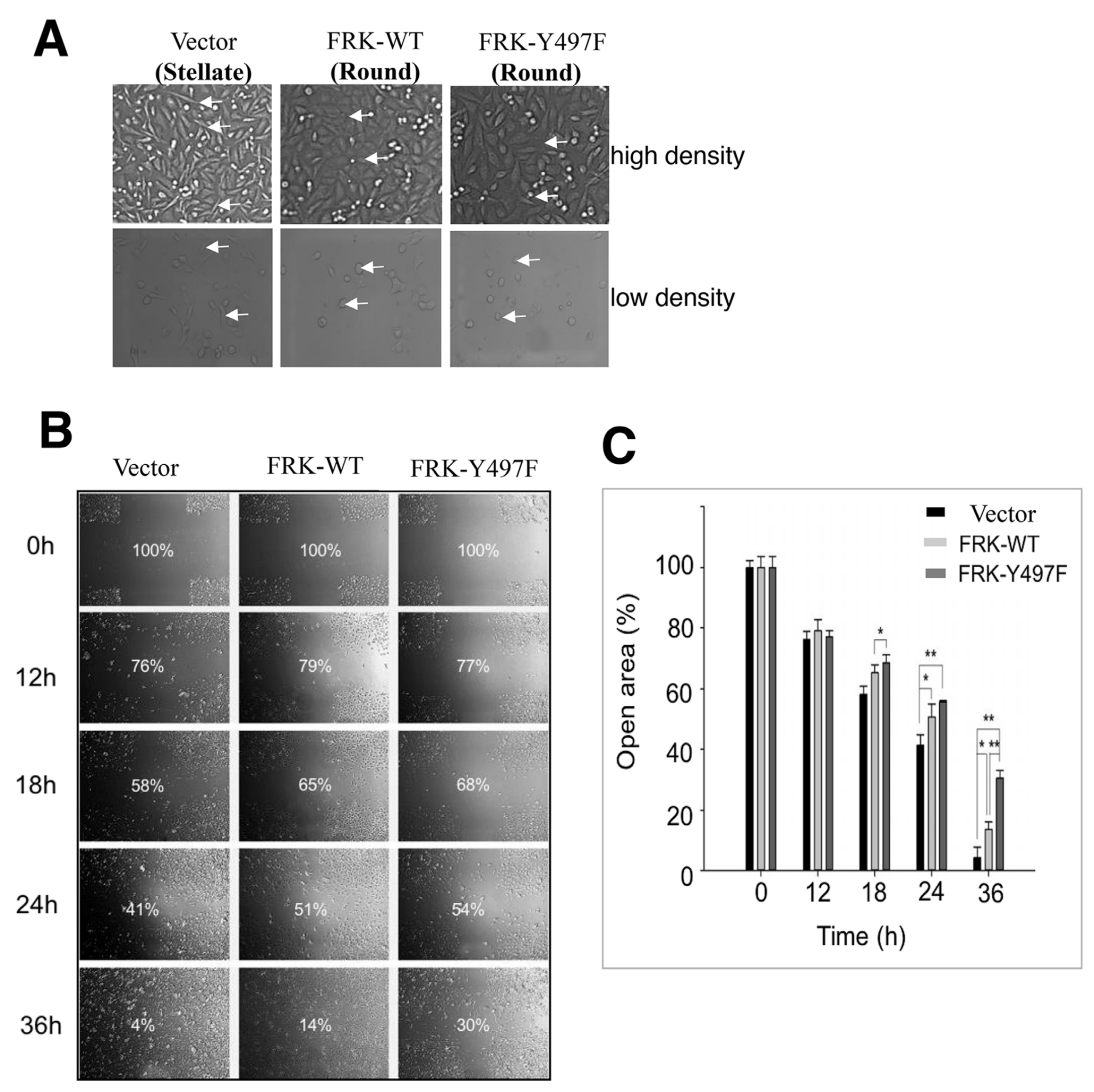

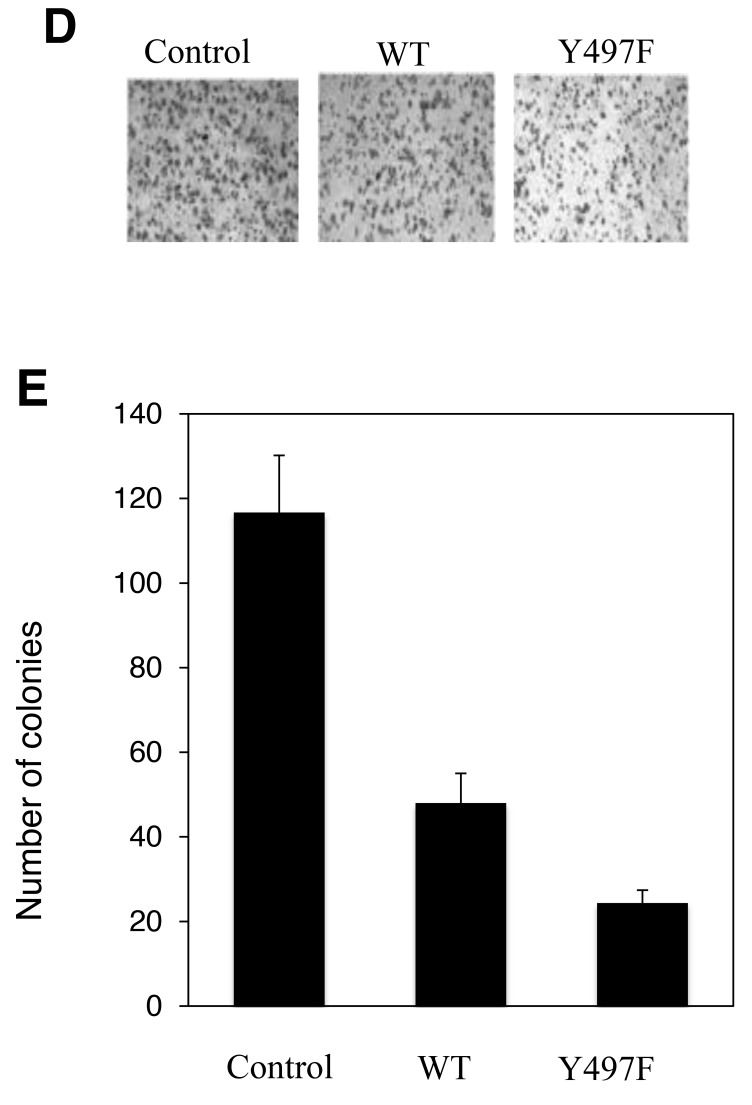
Effect of FRK expression on cell migration and invasion **(A)** The cellular morphology of the indicated stable cell lines was examined by microscopy.Arrows indicate the change from stellate to round shape. Phase-contrast images of the indicated stable cell lines were taken using the Olympus 1x51 microscope at 20x magnification. **(B)** Scratch-test assays were performed to determine the rate of cell migration in the indicated MDA-MB-231 stable cell lines. Phase-contrast images were taken at the indicated time intervals (0 hours-36 hours) using the Olympus 1x51 microscope. The progressive decrease in the percentage of open area, corresponding to each time interval, was calculated using the TScratch software (Ver 1.0). **(C)** Statistical analyses of the results from the scratch test were performed using the GraphPad PRISM software (Ver. 5.0) for all replicates (n=3). **(D)** Two-chamber Transwell invasion assays were performed to examine the effect of FRK on the invasive potential of the MDA-MB-231 cells *in vitro*. The empty vector control, Wild-type FRK (FRK-WT) and FRK Y497F stable cell lines were seeded in the upper chamber of 96-well plates and allowed to traverse to the chemoattractant in the bottom chamber. The number of cells migrated to the bottom chamber was manually counted following staining with crystal violet. **(E)** Statistical analyses of results from the replicate invasion assays (n=3) were performed using the GraphPad PRISM software (Ver 5.0) and presented as the relative number of visible cell colonies for each indicated cell line.

MDA-MB-231 are typically stellate-shape. However, we observed that the FRK-expressing cells lost the typical mesenchymal stellate morphology exhibited by the parental cells, and instead acquired a more rounded shape (Figure 5A). This distinct morphological change, which did not affect the total number of viable cells in all groups of cell lines studied (data not shown), was predicted to affect cell motility. We, therefore, investigated the effect of FRK on the migration of MDA-MB -231 stable cell lines using the wound-healing assay. Cross-shaped scratches were made to run perpendicular to each other across the diameter of 1 cm in 6-well plates seeded with MDA-MB-231 stably expressing FRK-WT, FRK-Y497F or the vector-transfected parental cells. As illustrated in Figure 5B and 5C, after 36 hours the open area of MDA-MB-231 cells stably expressing FRK-WT and FRK-Y497F were reduced to 14%, and 30%, respectively; while, that of the parental MDA-MB-231 was 4%. This implied that both stable cells lines had lower migration rates compared to the parental cell line (*P* < 0.05; Figure 5B and 5C). There was no significant difference in the cell migration rates between the parental MDA-MB-231 and MDA-MB-231 stably expressing the empty vector (data not shown). To further examine the effect of FRK on the invasive ability of breast cancer cells, a BD chamber coated with Matrigel was used. Our data showed that the number of cells invading the lower chamber was significantly reduced in the presence of FRK (*P* < 0.05; Figure 5D) by 35 % for FRK-WT and even more dramatically by 60 % in the FRK-Y497F (*P* < 0.05; Figure 5E). These results together suggest that FRK-WT and especially activated FRK-Y497F are effective in preventing breast cancer cell migration and invasion.

### FRK overexpression inhibits various JAK/STAT, MAPK, and Akt signaling pathways

**Figure 6:**
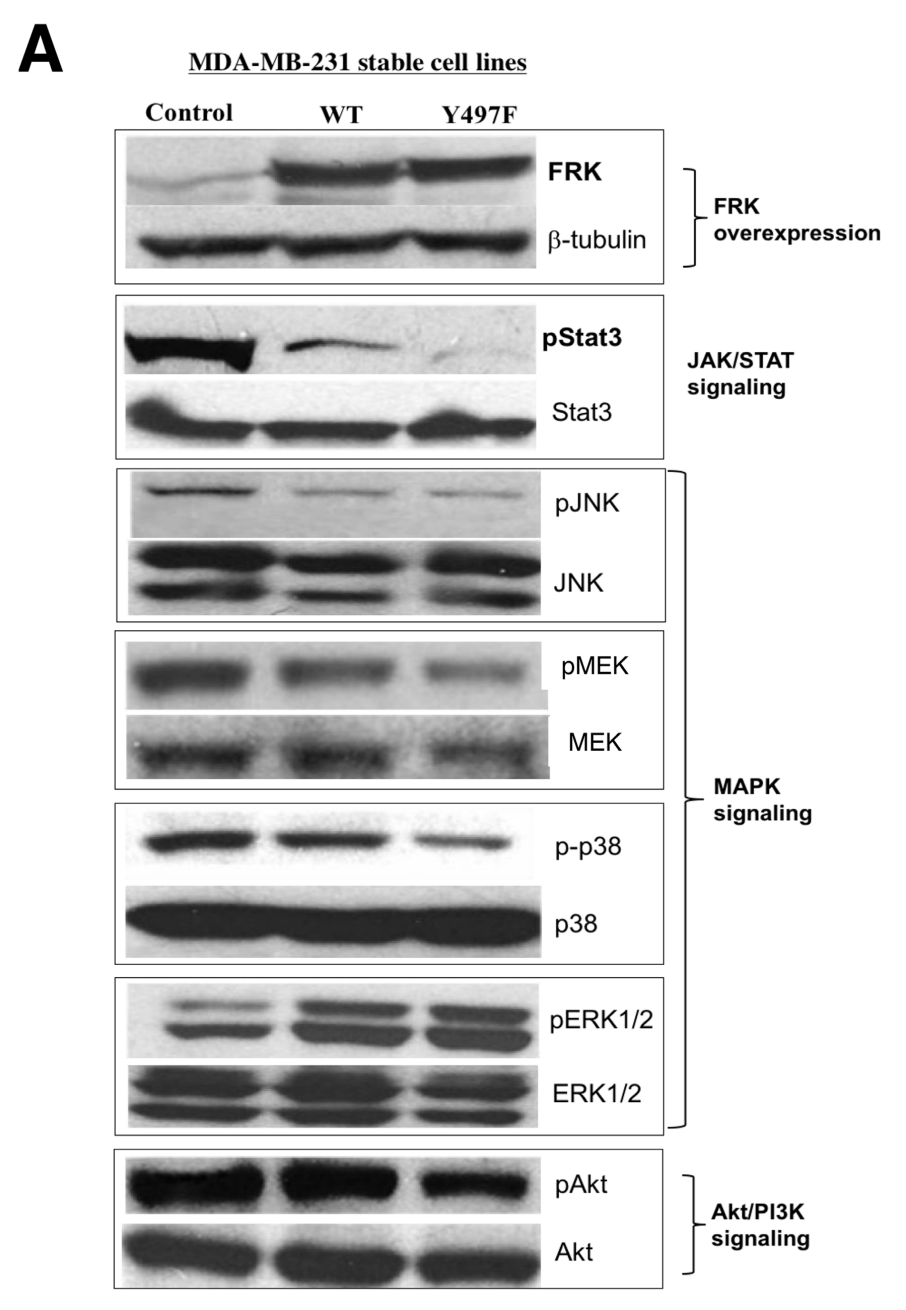

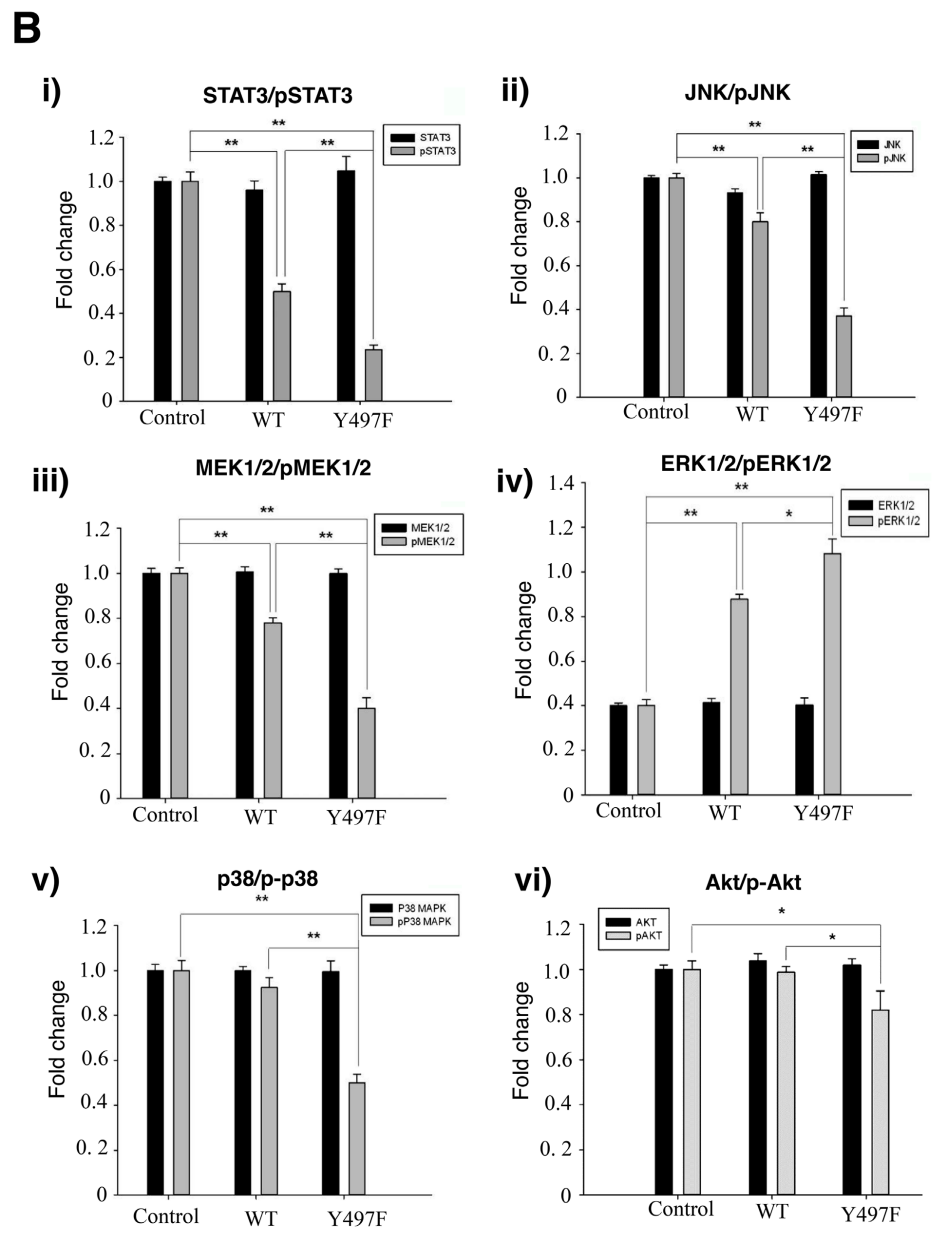
FRK-mediated regulation of signaling proteins in MDA-MB-231 cells **(A)** The empty vector control, Wild-type FRK (FRK-WT) and FRK Y497F stable cell lines were harvested, lysed and resolved via SDS-PAGE. Western blotting was performed using antibodies against the indicated signaling proteins corresponding to key signaling pathways. Antibodies against FRK was used to determine the expression of FRK in the stable cell lines. Beta-tubulin was used as the loading control. **(B) (i-vi)** Quantification of expression of the indicated proteins was performed using Image J (Ver. 1.48).

Several reports have shown that tyrosine kinases promote cell invasion and migration by EMT [26, 27]. Previous reports demonstrated that FRK suppresses cell proliferation in breast cancer by various mechanisms, which include the stabilization of PTEN and BRCA1 and the internalization of EGFR [8, 14, 15]. Also, FRK was shown to suppress glioma cell migration and invasion by regulating JNK/c-Jun signaling and promoting N-cadherin/β-catenin complex formation [10, 12]. We have shown that FRK also suppresses cell migration and invasion of breast cancer cells. To determine the mechanism by which FRK regulates breast cancer cell migration and invasion, we employed kinome assays to identify potential FRK-regulated signaling pathways. We used a well-characterized kinome array comprising 300 different target peptides, derived from various signaling pathway mediators including those from the PI3K, JAK-STAT, and MAPK signaling pathways [30, 33]. We analyzed lysates from HEK-293 cells transiently transfected with FRK-WT and the parental cells. We observed that the presence of FRK either enhanced/promoted or downregulated the phosphorylation of target peptides on the array. The majority of FRK-regulated peptides were hypophosphorylated relative to the control. These included peptides derived from the JAK/STAT signaling intermediates such as JAK1, BCL2, JNK1, STAT1, and STAT3, as well as Akt and MAPK signaling pathways (Supplementary Table 4). Hyperphosphorylated peptides were fewer and included those derived from CRK, GRB10, and GRB2. To validate our kinome data, we evaluated the phosphorylation status of selected targets and some of their related signaling partners by immunoblotting analysis using phospho-specific antibodies in our MDA-MB-231 stable cell lines. The antibodies used included those against Akt, p-Akt, MEK1/2, phospho-MEK1/2, P38 MAPK, p-p38 MAPK, JNK, p-JNK, STAT3, p-STAT3, FRK and β-Tubulin as the control. As shown in Figure 6A, FRK regulated the JAK/STAT, MAPK, and Akt signaling pathways. Specifically, the presence of FRK resulted in a significant suppression of the constitutive phosphorylation of STAT3, JNK, Akt, and MEK1/2. Surprisingly, we observed an increase in the activation of ERK1/2 (Figure 6A). These experiments were repeated at least three times and quantified with the reproducible outcome (Figure 6B, i-vi). The most dramatic effect of FRK was observed with STAT3 where the presence of either FRK-WT or FRK-Y497F led to the inhibition of the constitutive activation of STAT3 by 2 and 5-fold, respectively. Although, Pilati *et al*. showed that mutation of FRK kinase domain increased FRK kinase activity and STAT3 phosphorylation [6]. However, we did not see any effect on STAT3 activation when the same mutation was introduced into FRK kinase domain and transfected in breast cancer cells (Supplementary Figure 1). Our data suggest that FRK could promote the inhibition of cell growth and migration by suppressing the activation of various signaling pathways, and more significantly the STAT3 signaling pathway and the tumor suppressor role of FRK could be tissue dependent.

### FRK suppresses the expression of STAT3 target genes in breast cancer cell lines

**Figure 7:**
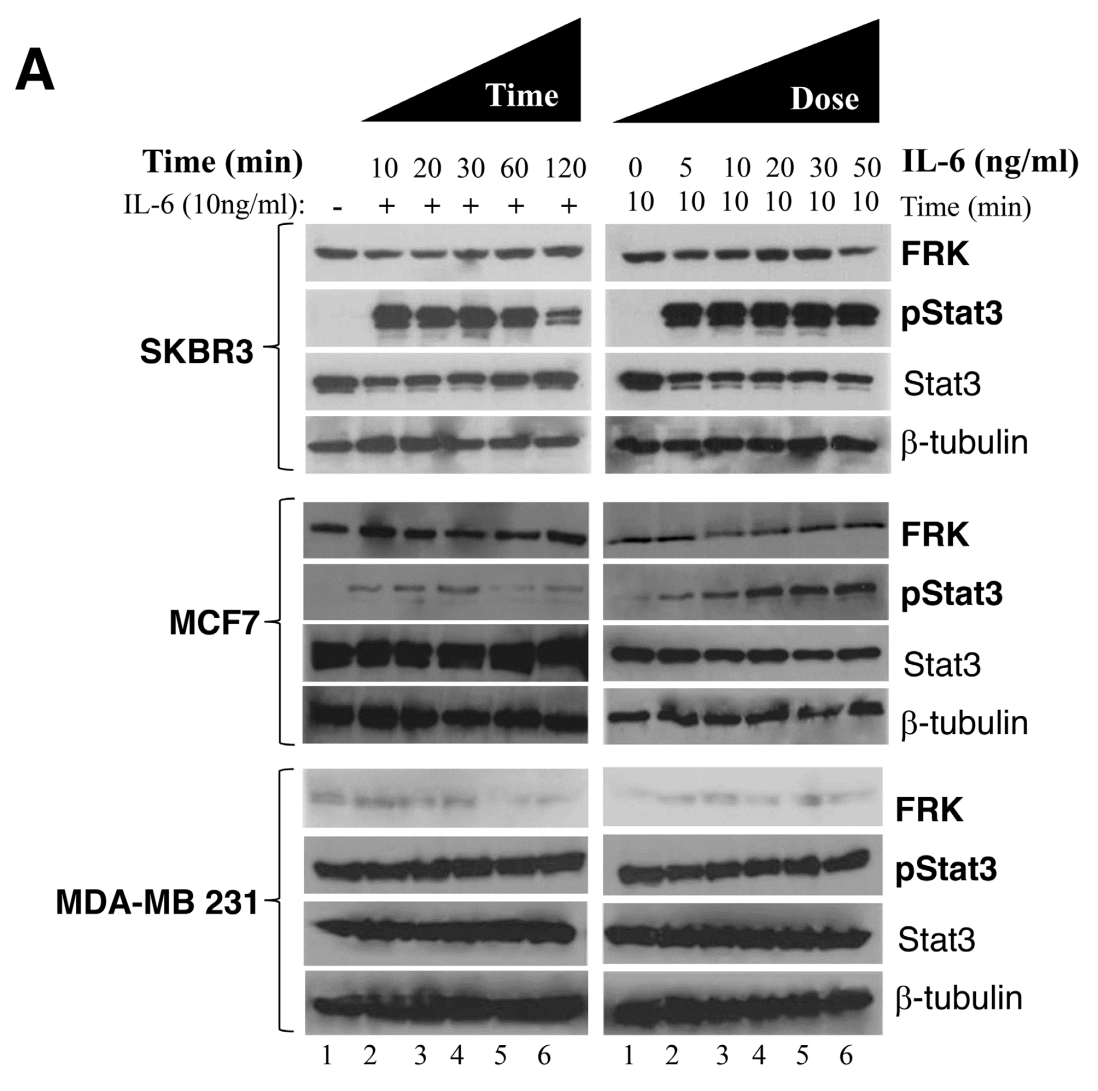

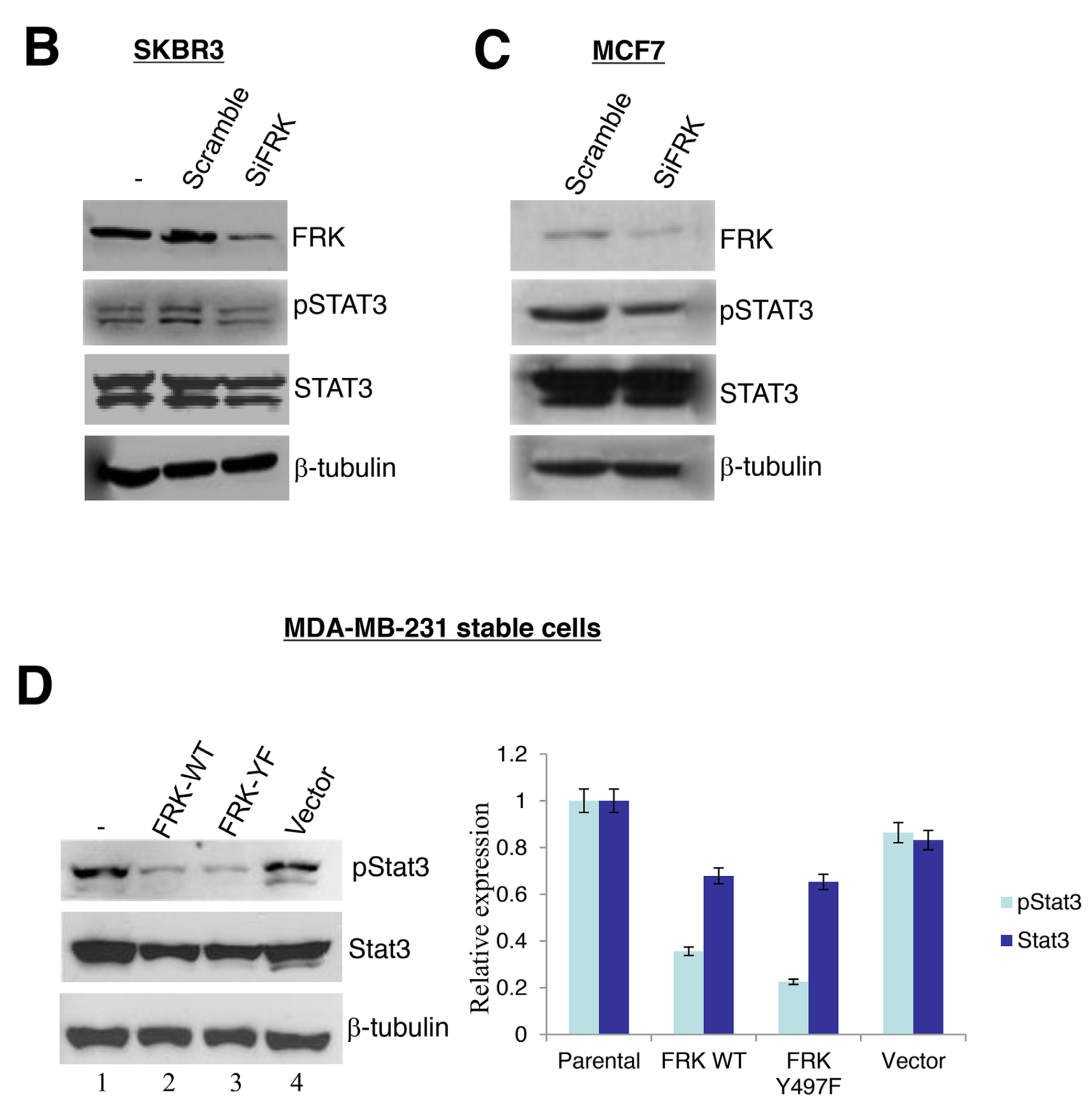
Effect of FRK overexpression and knockdown on STAT3 phosphorylation **(A)** IL-6-induced activation of STAT3 in FRK stable cell lines. Parental SKBR3, MCF7 and MDA-MB-231 cells were seeded in 6-well plates and stimulated with 10ng/mL IL-6 for the indicated time points (left panel). Cells were subsequently harvested, lysed and total proteins resolved via SDS-PAGE. Western blotting was performed using the antibodies against total and phospho-STAT3, FRK and beta-tubulin (loading control) (left panel). Next, the IL-6 dosage was empirically determined for the same cell lines (right panel) using a fixed treatment time of 30 minutes. Cells were lysed, and Western blotting carried out using the same antibodies described above (right panel). **(B and C)** Endogenous FRK were transiently knocked down in the SKBR3 and MCF7 cell lines. The efficiency of FRK knockdown and its effect on STAT3 phosphorylation was determined by Western blotting using antibodies against FRK (right-hand panel). Beta-tubulin was used as the loading control. Parental cells were used as the reference. The level of total and phospho-STAT3 in the parental, scramble siRNA and FRK siRNA-transfected cells were determined by Western blotting using relevant antibodies **(D)** The effect of FRK overexpression on STAT3 phosphorylation. Parental MDA-MB 231 or control, FRK WT and FRK YF stable MDA-MB 231 cells were lysed, and Western blotting performed to determine the levels of total and phospho-STAT3. Beta-tubulin was used as the loading control. **(E and F)** Quantification of expression of the indicated proteins was performed using Image J (Ver. 1.48) and ratio of pSTAT3: STAT3 to determine the activity of pSTAT3 in the cell lines were quantified.

STAT3 signaling has been well characterized in breast cancer cells [34–37]. It is known for its role in tumor cell growth, migration, invasion, and metastasis [38]. We opted to further characterize the effect of FRK on STAT3 signaling in breast cancer cells. Time- and dose-dependent stimulation of these cells with IL-6 resulted in a corresponding increase in STAT3 activation, MCF-7, and SKBR3 cells were all sensitive to IL-6 stimulation, with exception to MDA-MB -231 (Figure 7A). However, when we tested the effect of IL-6 stimulation on STAT3 activation status in MDA-MB-231 transiently expressing FRK-WT, FRK-Y497F or FRK-K262M. We found that FRK-Y497F decreased the level of IL-6-stimulated activation of STAT3 (Supplementary Figure 6). Next, we transiently depleted FRK from FRK-positive breast cancer cell lines, SKBR3 and MCF-7 by RNAi. We obtained about 70% knockdown of endogenous FRK in SKBR3 cells (Figure 7B) compared with the levels in the parental cell line and the scrambled control lysate. Next, we evaluated the effect of FRK knockdown on STAT3 activation by immunoblotting using antibodies against STAT3 and pSTAT3 (pY705). Interestingly, FRK knockdown did not affect STAT3 activation in SKBR3 (Figure 7B). We also depleted FRK by about 70% in MCF-7 cells and observed no significant effect on STAT3 activation (Figure 7C). However, since we consistently observed a strong inverse correlation between STAT3 activity and FRK overexpression in MDA-MB-231 as reproduced in Figure 6A and 6B, and Figure 7D, 7E, and 7F, we investigated the effect of FRK on STAT3-regulated genes.

**Figure 8:**
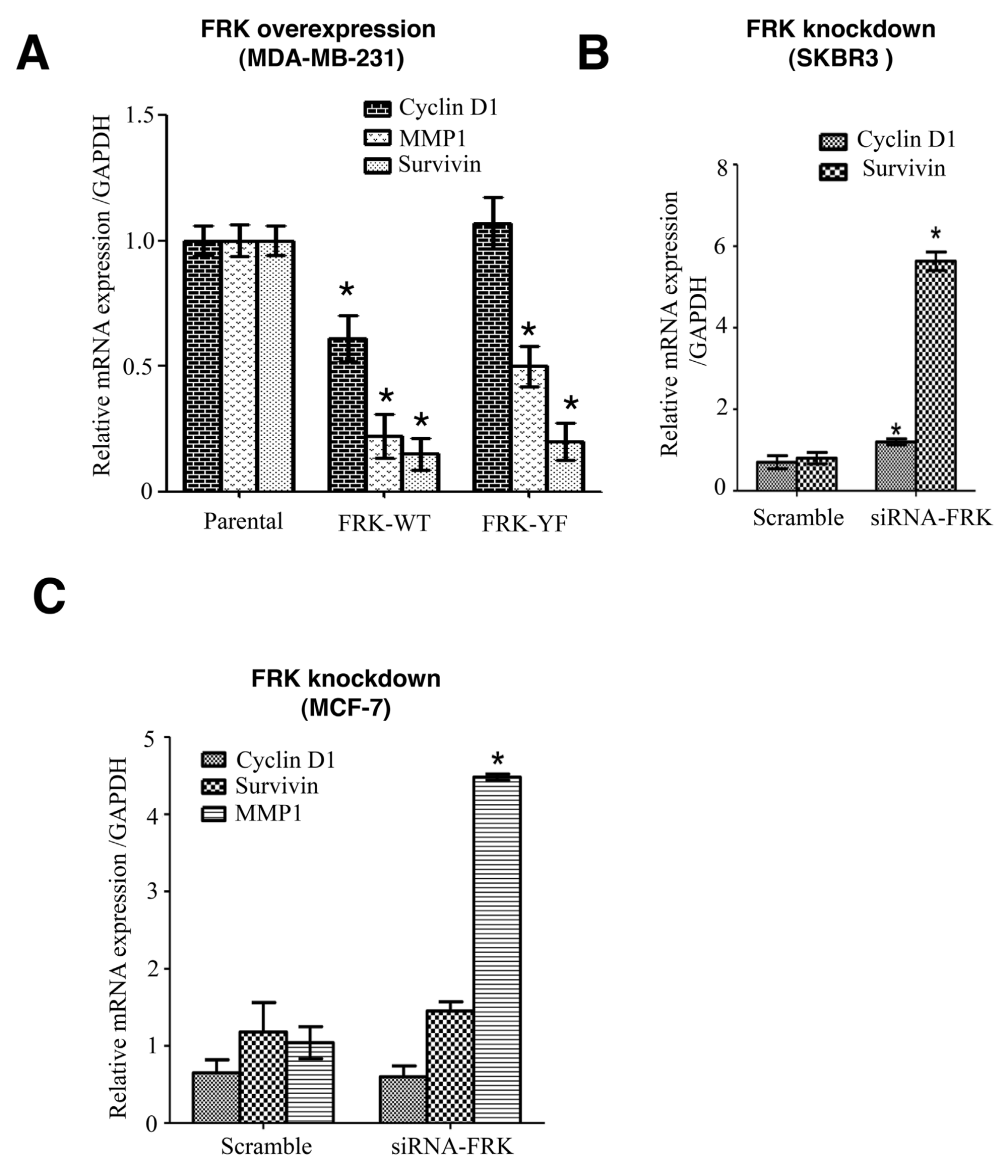
The effect of FRK overexpression and knockdown on STAT3-target genes expression **(A)** The mRNA levels of Survivin, Cyclin D1, and MMP1 were quantified via quantitative RT-PCR in the empty vector control, FRK WT and FRK-Y497F MDA-MB 231 stable cell lines. **(B and C)** The mRNA levels of Survivin, Cyclin D1 and MMP1 in SKBR3 and MCF7 cells were quantitatively measured relative to the parental cells using a via quantitative RT-PCR.

Many target genes of STAT3 are relevant to various human cancers where they play pivotal roles in many cellular processes including tumor growth and progression [39]. We reasoned that the association between FRK expressions and STAT3 activation status might have a causational effect on STAT3 target genes. These genes include Survivin, cyclin D1 and matrix metalloproteinase 1 (MMP-1). We performed RT-PCR to determine the expression levels of selected STAT3 target genes in FRK-overexpressing and the knockdown breast cancer cell lines. We observed that MDA-MB-231 cell lines stably overexpressing FRK-WT displayed reduced levels of STAT3 target genes Survivin, cyclin D1 and MMP-1 (Figure 8A). Although a similar observation was made for FRK-Y497F stable cells, we found that FRK-YF increased cyclin D1 gene expression, we are not sure why this mutant increased cyclin D1 expression (Figure 8A). These data show that FRK inhibition of STAT3 activation results in the impairment of STAT3 downstream signaling events. Although FRK knockdown had little effect on STAT3 activation in SKBR3 and MCF-7 breast cancer cells, we noted that the knockdown of FRK led to the upregulation of Survivin mRNA levels in both cell lines (Figure 8B and 8C). Cyclin D1 and survivin levels were also upregulated in SKBR3 (Figure 8B), MMP-1 was not in SKBR3. However, MMP-1 mRNA levels were increased in FRK-knockdown MCF-7 cells (Figure 8C), there was no significant effect of FRK knockdown on cyclin D1 and survivin in MCF-7. Together our data indicated that FRK overexpression decrease STAT3 phosphorylation and some of its downstream target genes. Although we obtained no effect of FRK knockdown on STAT3 phosphorylation, we observed an increase in STAT3 target genes with FRK knockdown. Our results show that FRK might regulate the genes through other mechanisms other than STAT3 signaling pathway.

### FRK- regulates EMT in breast cancer cells

**Figure 9:**
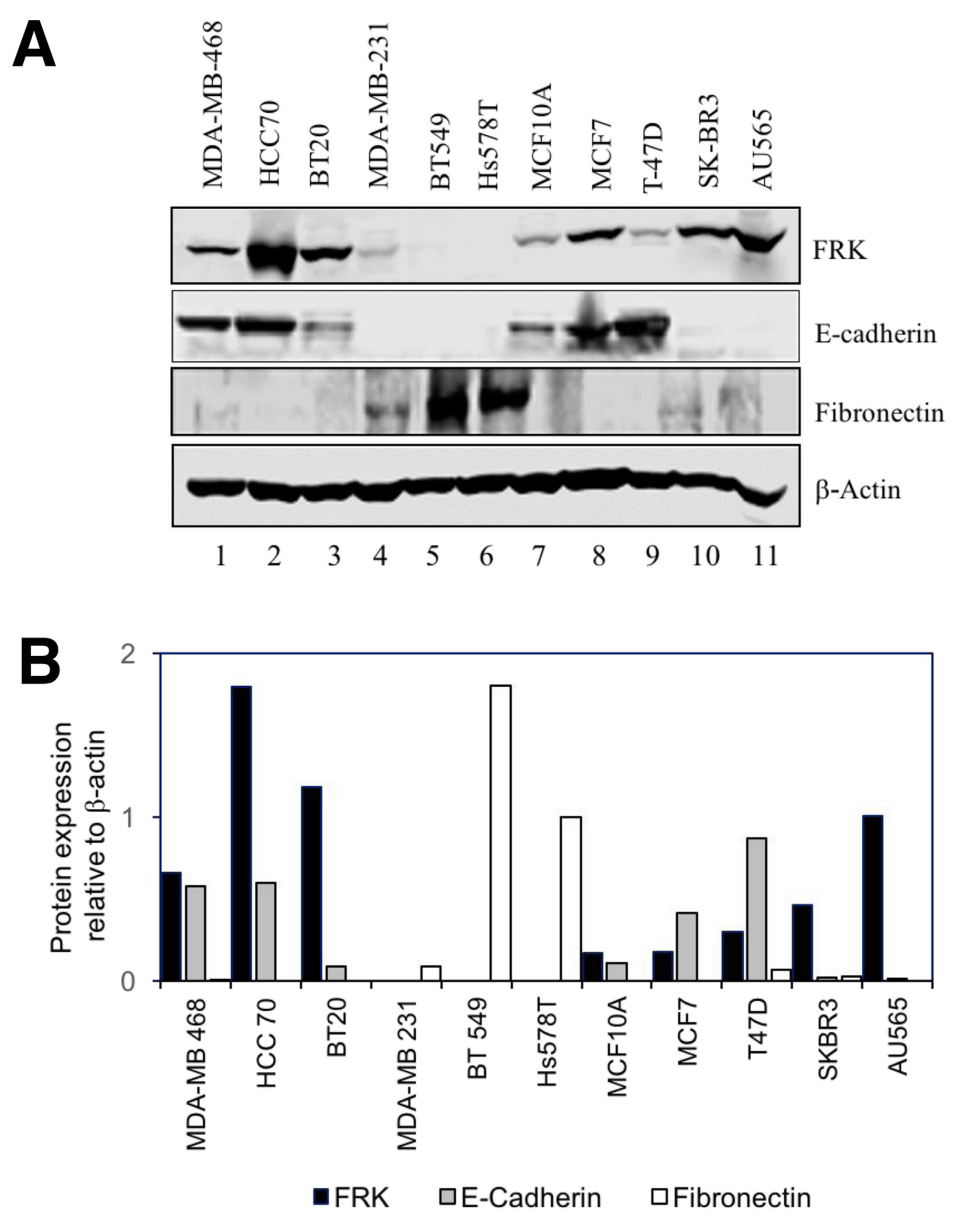

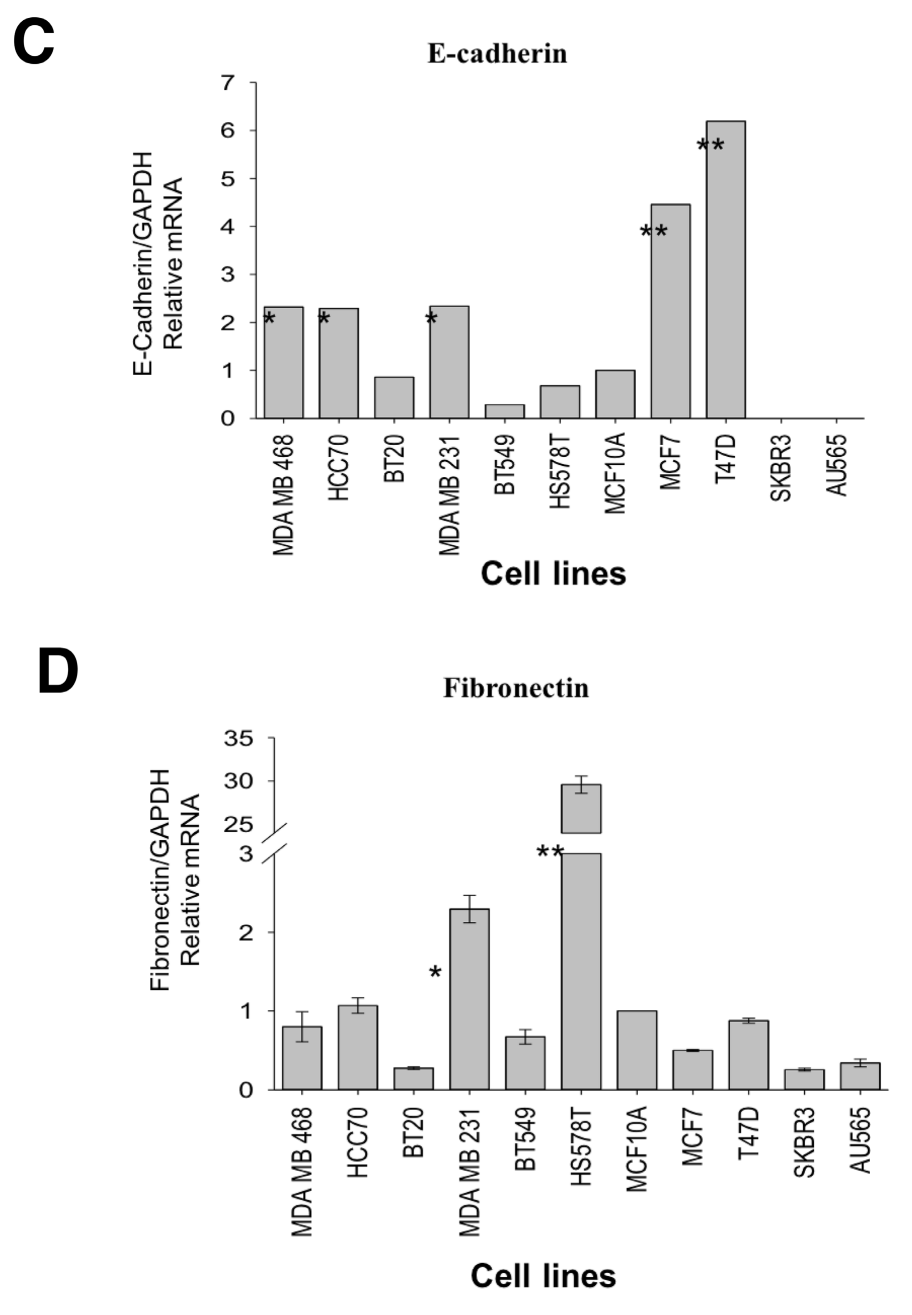

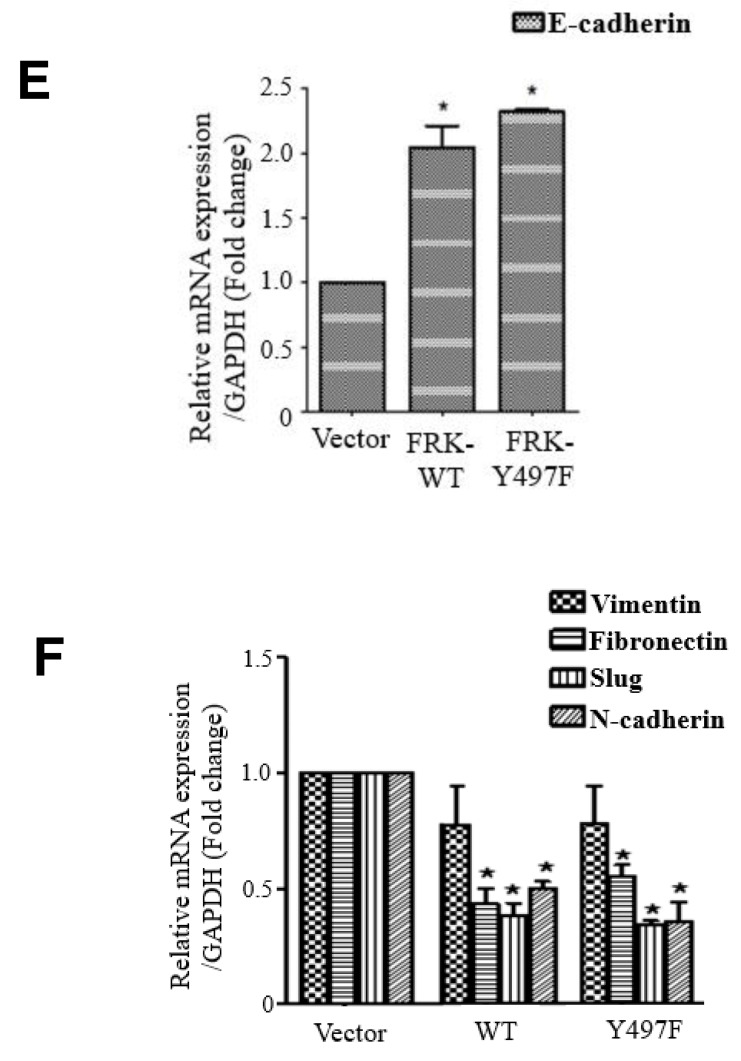

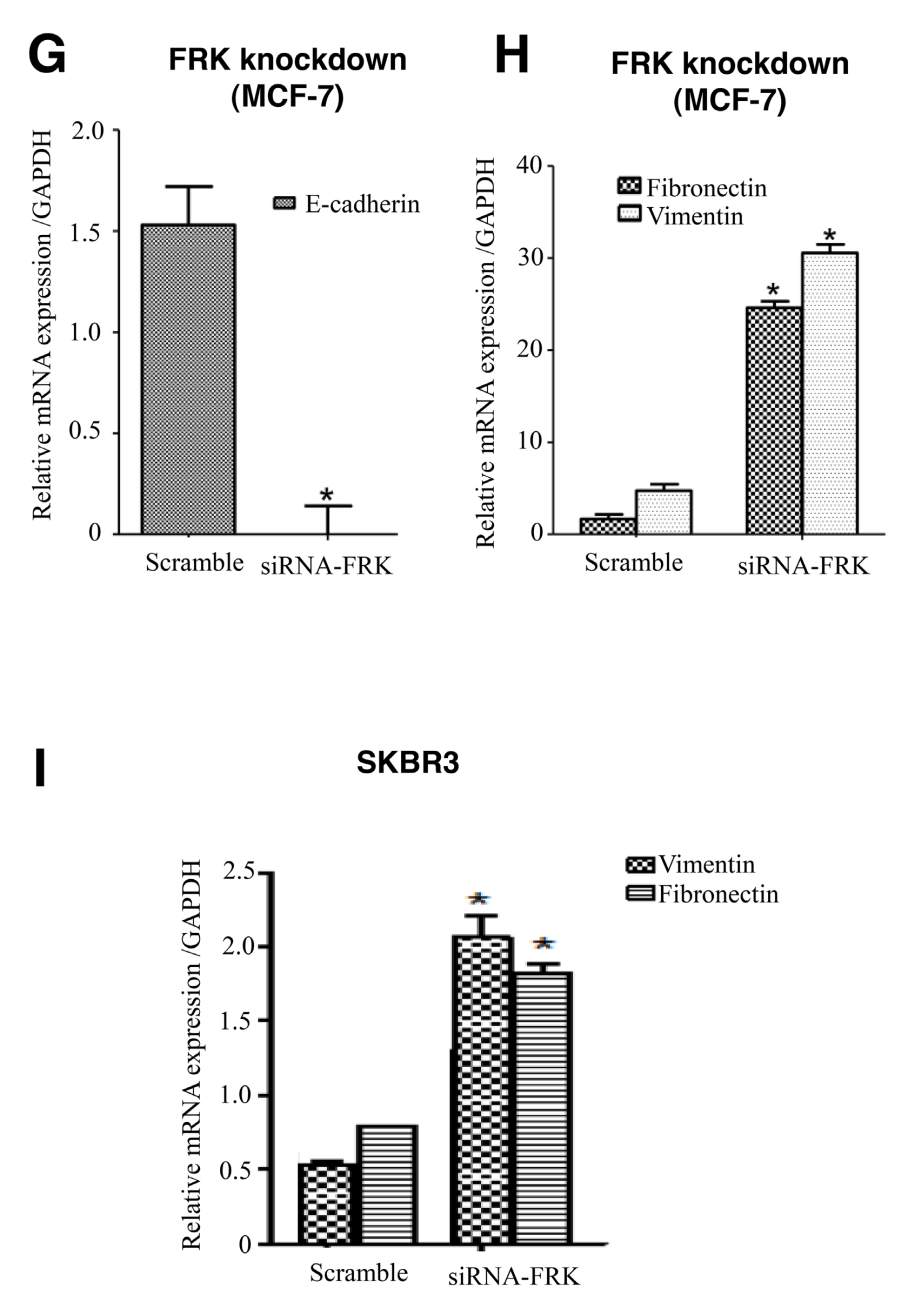
FRK-mediated regulation of EMT in breast cancer cell lines **(A)** The expression of E-Cadherin and Fibronectin was examined by a selected panel of breast cancer cell lines and the MCF10A cell line using appropriate antibodies. Beta-actin was used as the loading control. **(B)** The protein expression levels were quantified using the Image J software (Ver. 1.48) and presented as fold-change expression relative to beta-actin. **(C)** and **(D)** mRNA levels of E- cadherin, and Fibronectin relative to GAPDH, were quantitatively measured via RT-PCR. ^*^ represents statistical significance of p≥0.05 and ‘^**^’ represent statistical significance of p≥0.001. **(E and F)** The mRNA levels of E-Cadherin, Vimentin, Fibronectin, Slug, and N-Cadherin, relative to GAPDH levels, were quantitatively measured in the control vector, FRK WT and FRK Y497F-expressing MDA-MB 231 stable cell lines. ^*^ represents statistical significance of p≥0.05. **(G and H)** Endogenous FRK was transiently knocked down in MCF7 cells and the mRNA levels of E-Cadherin, Fibronectin and Vimentin were quantified relative to GAPDH transcript levels. Parental MCF7 cells were used as the reference. ^*^ represents statistical significance of p≥0.05. **(I)** The mRNA levels of Vimentin and Fibronectin were quantitatively measured in SKBR3 cells following the transient knock-down of endogenous FRK. Parental SKBR3 cells were used as the reference.

FRK have been shown to suppress EMT markers in glioma cells [10]. Our results in Figure 1,show that FRK protein and mRNA expression was moderate/high in Luminal and Basal A cells that display an epithelial-like phenotype, and low or undetected in Basal B cells which are more mesenchymal in nature. Further, we detected the expression of FRK predominantly in the epithelial layer lining the mammary ducts of the normal breast tissue (Figure 2). Based on these observations, we hypothesized that FRK might regulate EMT in breast cancer cells. Thus, we examined the effect of FRK on the expression of well-known epithelial marker E-cadherin, and mesenchymal markers such as vimentin, fibronectin, slug and N-cadherin in breast cancer cells. First, we determined the protein and mRNA expression of E-cadherin and fibronectin across 11 breast cancer cell lines. Interestingly, we found the Basal A and Luminal breast cancer cells express moderate protein levels of E-cadherin, (an epithelial marker) and low/undetectable amount of fibronectin (a mesenchymal marker) (Figure 9A and 9B). On the other hand, mesenchymal-like/Basal B breast cancer cells expressed high/moderate protein levels of fibronectin with a little/undetectable expression of E-cadherin cells (Figure 9A and 9B). This result was consistent with the expression levels of N-cadherin in these cells (Data not shown). We next examined the mRNA levels of these markers in all 11 cell lines. Consistent with the protein data, our results in Figure 9C demonstrate a higher level of expression of E-cadherin mRNA in the Basal A and Luminal cells compared with Basal B. Although the levels of fibronectin mRNA were generally low in most of the cell lines, a significant spike in the expression was detected in Hs587T cells, a Basal B/mesenchymal-like cell line (Figure 9D). This observation was consistent with the mRNA expression mined from the Gene Expression Omnibus (https://www.ncbi.nlm.nih.gov/geo/) (Supplementary Figure 7). Taken together, our data demonstrate that FRK inversely correlates with mesenchymal markers in breast cancer cells and may, therefore, be a negative regulator of mesenchymal-like properties of breast cancer cells.

To directly determine the effect FRK on EMT in breast cancer cells, we examined the expression of epithelial and mesenchymal markers in the MDA-MB 231 cells stably expressing either FRK-WT or FRK-Y497F, as well as in FRK knockdown SKBR3 and MCF 7 cells via Real-time PCR and Western blotting analyses. Overexpression of both FRK-WT and Y497F in MDA-MB-231 cells significantly upregulated the levels of E-cadherin mRNA (Figure 9E) and downregulated several mesenchymal markers such as slug, fibronectin, and N-cadherin at the mRNA (Figure 9F). Although the presence of FRK-WT or Y497F dramatically reduced the levels of fibronectin and N-cadherin protein, no change in slug protein levels was observed (Supplementary Figure 8). We also observed a decrease in menschenmyal markers such as fibronectin in Hs578T breast cancer cell line with the transient overexpression of FRK (Supplementary Figure 9A and 9B). We also saw a minor increase in E-cadherin protein level in the presence of FRK-WT or Y497F (Supplementary Figure 8). The knockdown of FRK in MCF-7 resulted in a significant increase in vimentin and fibronectin mRNA levels, and a decrease in E-cadherin mRNA levels (Figure 9G and 9H). There was a corresponding increase in the protein levels of fibronectin and slug. However, there was no significant effect of FRK knockdown on E-cadherin protein levels (Supplementary Figure 8). The knockdown of FRK in SKBR3 also resulted in a significant increase in vimentin and fibronectin mRNA levels (Figure 9I), but its effect on E-cadherin mRNA and protein expression could not be analyzed because SKBR3 does not express E-cadherin [20]. Together, our data consistently showed that the overexpression of FRK increased the expression of E-cadherin mRNA and down-regulated the transcript levels of fibronectin, N-cadherin and slug, while knockdown of FRK in both MCF-7 and SKBR3 cells led to the upregulation of vimentin and fibronectin mRNA levels. Our findings, therefore, suggest that FRK inhibits EMT by stimulating the expression of the epithelial marker, E-cadherin and suppressing the expression mesenchymal proteins, especially vimentin and fibronectin.

### FRK expression inversely correlates with mesenchymal markers in breast cancer cell line

**Figure 10:**
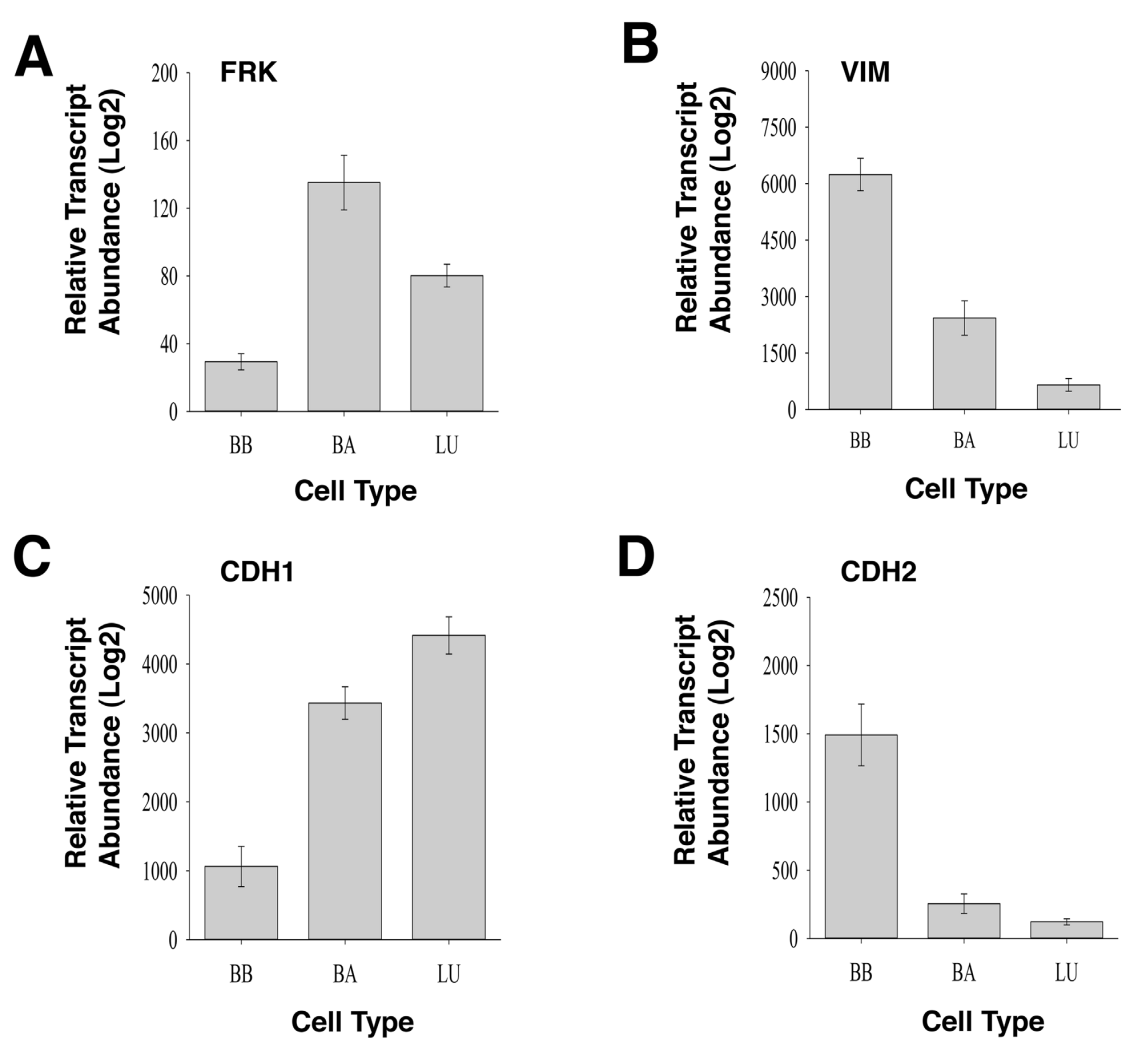

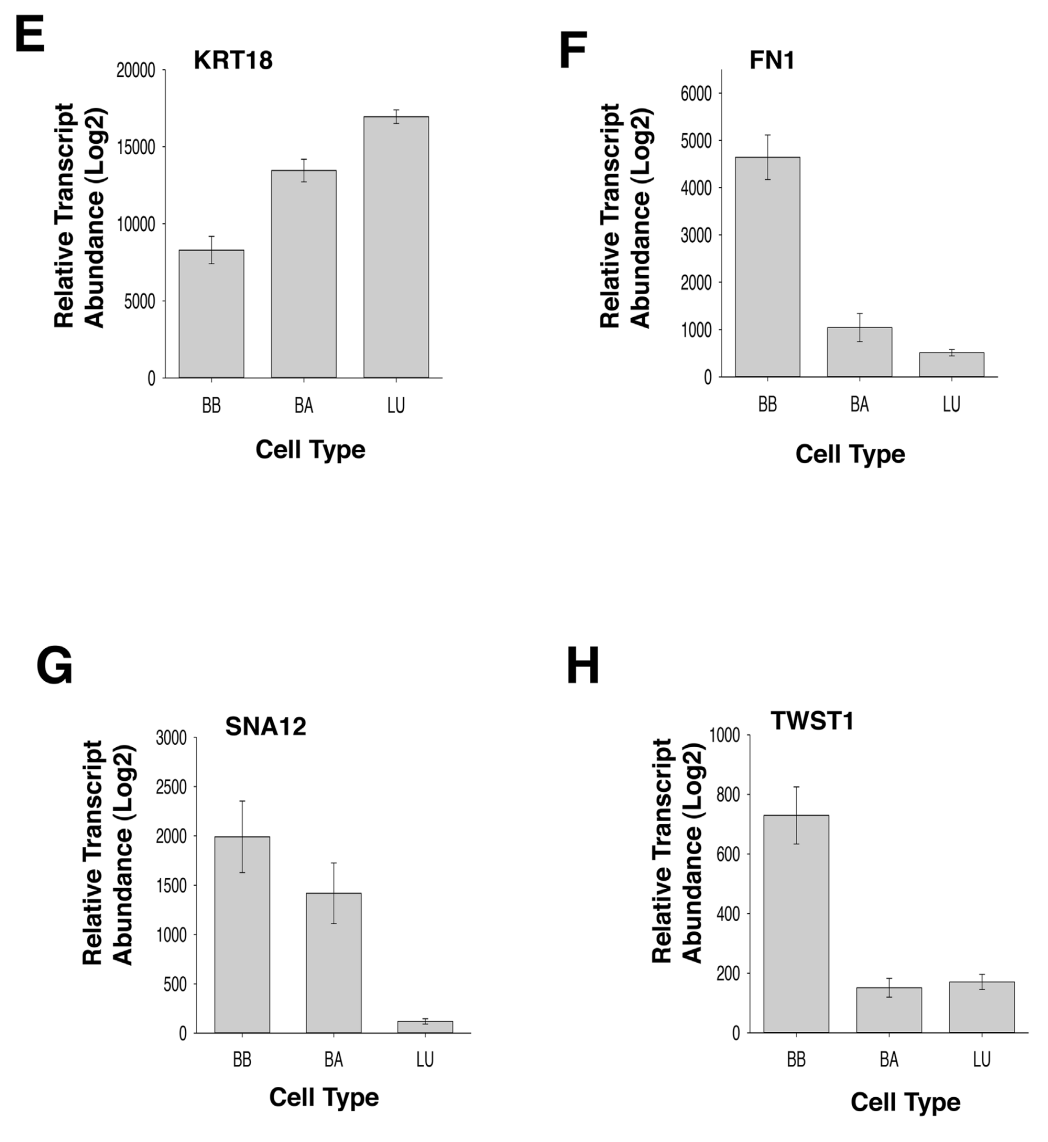
Gene expression profile of FRK and epithelial and mesenchymal markers in Basal A, Basal B, and luminal breast cancer cell lines Logarithmized transcript abundances of **(A)** FRK, **(B)** Vimentin, VIM; **(C)** E-cadherin, CDH1; **(D)** N-cadherin, CDH2; **(E)** Keratin 18, KRT18; **(F)** Fibronectin, FN1, **(G)** Slug, SNAI2 and **(H)** TWIST1 were assessed in Basal A (BA), Basal B (BB) and Luminal (LU) breast cancer cell lines (N=56). Mean transcript levels of the indicated genes in breast cancer cell lines were mined from the GSE10021, GSE10843, GSE3156, GSE10890 and GSK's cell line project (https://array.nci.nih.gov/caarray/project/woost-00041/) databases using the GENT software (http://medicalgenome.kribb.re.kr/GENT/reference.php). The data are presented as mean ± SEM. (p-value ≤ 0.05).

To substantiate our findings that FRK is a negative regulator of EMT, we mined the gene expression database, GENT (Gene Expression across Normal and Tumors; http://medicalgenome.kribb.re.kr/GENT/reference.php) to determine the correlation between FRK expression and epithelial and mesenchymal markers. We examined the relationship between the expression of *FRK* with mesenchymal markers Vimentin (*VIM*), N-cadherin (*CDH2*), fibronectin (*FN1*), snail family transcriptional repressor 2 (*SNAI2*), twist family bHLH transcription factor 1(*TWIST1*), and epithelial markers E-cadherin (*CDH1*) and Keratin 18 (*KRT18*), in breast cancer cell lines stratified under Basal B (BB), Basal A (BA) and luminal (LU) (Supplementary Table 5, Figure 10). We recently reported the level of FRK transcript was low in the basal B breast cancers as compared to Basal A and Luminal cells [40]. We found that the mean transcript expression of *VIM*, *CDH2*, *FN1*, and *TWIST1* were higher in the basal B breast cancer cells with low *FRK* transcript levels as compared to Basal A and Luminal cells that harbor high *FRK* transcript levels (P<0.05; Figure 10B, 10D, 10F and 10H). Mean transcript levels of *SNAI2* were higher in the basal B breast cancer cells with low *FRK* transcript levels as compared to the Luminal cells (P<0.05; Figure 10G). Pearson's correlation analysis run on 226 samples made up of 56 breast cancer cells showed that the *FRK* transcript levels negatively correlated with the transcript levels of *VIM*, *CDH2*, and *TWIST1*, with R-values of -0.28; -0.20; -0.25 respectively (P<0.001). The mean transcript expression of CDH1 and KRT18 were lower in the basal B breast cancer cells with low *FRK* transcript levels as compared to Basal A and Luminal cells that harbor high FRK transcript levels (P<0.05; Figure 10C, 10E). *FRK* transcript levels correlated positively with the transcript levels of *CDH1* and *KRT18*, with R-values of 0.39 and 0.26 (P<1.0 × 10^−5^), respectively. Although this trend was not completely reciprocated when we interrogated the cancer genome atlas (TCGA) dataset (http://cancergenome.nih.gov/), a positive correlation was also observed with E-cadherin, Fibronectin, and Vimentin in normal tissues samples (Supplementary Figure 10). However, no correlation was observed between FRK and Vimentin, E-cadherin and Fibronectin in the tumor samples (Supplementary Figure 10). The TCGA breast carcinoma dataset, unlike the information on cell lines, is not classified by Basal A/Basal B mesenchymal properties, and this may explain the discrepancy in both datasets. However, the results obtained in Figure 10 suggests that FRK expression inversely correlates with mesenchymal markers in Basal B breast cancers cells and present a contextual nature for FRK in EMT-associated cellular processes.

## DISCUSSION

FRK is a member of the BRK family kinases [1, 2]. It is a Src-related tyrosine kinase that is structurally similar to SFKs except for its lack of an amino-terminal myristoylation site [1]. Unlike most SFKs and BRK, which tend to display cell growth-promoting properties and have varying levels of transforming activity [2, 32], FRK has been characterized as a putative tumor suppressor in various cancers including breast cancer and glioma where it has growth inhibitory, rather than transforming activity [1, 10–12, 14, 41]. FRK was found to be expressed predominantly in mammary epithelial cells [4, 42]; however, its correlation to breast cancer cell subtype or stemness has not been reported.

Breast cancer is a heterogeneous disease with multiple criteria for classification based on clinical, histopathological markers and gene expression profiling [17–19, 43, 44]. Some of the breast cancer subtypes include luminal A, luminal B, basal-like and HER2-positive [17–19, 43, 44]. Basal breast cancer cell lines have been subdivided into basal A and basal B sub-categories [45, 46]. Basal A cell lines are associated with the up-regulation of several genes in the E-twenty-six transformation-specific (ETS)-pathway and mutations of the tumor suppressor genes BRCA1 and 2; while the basal B cell lines are Claudin-low and display mesenchymal and stem cell-like characteristics [45, 46]. Previous studies on the role of FRK expression in breast cancers were based on molecular subtypes of breast cancers. We investigated the expression pattern of FRK in breast cancer cell lines classified based on their morphology and invasiveness. We found that the expression of FRK was high in epithelial-like breast cancer cell lines and the normal mammary tissue, and is low or lost in in basal B breast cancer cell lines which display a mesenchymal phenotype. Our study provides experimental evidence for a potential role of FRK in the maintenance of the normal epithelium.

It was previously reported that FRK acts as a tumor suppressor in breast cancer where it was shown to inhibit cell growth and suppresses tumorigenesis. For example, the growth of FRK over-expressing MCF-7 breast cancer cells was significantly inhibited, while the loss of FRK in normal mammary epithelial cells induced tumor formation [8]. Since FRK is lost in Basal B cells including the MDA-MB-231 cell line, we used these cells to generate cell lines that stably expressed the wild-type protein and its constitutively active variant. Our goal was to evaluate the effect of FRK wild-type and the constitutively active form of the kinase on several cellular processes. No previous studies have examined the effect of FRK activation on its tumor suppressor function. We have previously shown that the constitutively active variant of BRK significantly promoted the oncogenic properties of the enzyme [28, 31]. Stable overexpression of FRK in the Basal B/FRK-negative MDA-MB-231 breast cancer cell line resulted in reduced cell proliferation, migration, invasion, and colony formation. Although, Meyer and his group observed an increase in cell proliferation when FRK-Kinase-defective mutant was transfected in BT474 breast cancer cell [41]. We, however, obtained no significant increase in cell prolif eration when FRK-KM (kinase-defective) mutant was transiently transfected into T47D breast cancer cell line (Supplementary Figure 2G). It is possible to see an effect on cell proliferation if FRK-KM was stably knocked down in T47D breast cancer cells. Although we have not investigated the mechanism by which FRK inhibits cell proliferation, it is worth mentioning that FRK has been showed to inhibit cell proliferation via inducing G1 arrest of the cell cycle [11, 41]. We hope to further investigate this in future. Overall, our data implied that activation of FRK was important but not essential for the tumor suppressor activity of the kinase.

Kenny *et al*. classified breast cancer cell lines into four distinct morphological groups [47]. These groups were denoted as Round, Mass, Grape-like, and Stellate. MDA-MB-231 was classified on the stellate group that also included BT-549. MDA-MB-436 and Hs578T. The Mass class usually display disorganized nuclei and include cell lines such as BT-474, HCC70, MCF-7, and T-47D. The Round cell class comprised of HCC1500, MCF-12A, and MDA-MB-415. The Grape-like class included cell lines such as AU565, MDA-MB-468, and SK-BR-3, characterized by poor cell-cell interaction. The Stellate class cells are distinctively more invasive than members of the other three groups, and the stellate projections tend to bridge multiple colonies of cells [47]. We noted in Figure 1A of the present study that all three stellate-shaped cell lines (MDA-MB-231, BT-549, and Hs578T) did not express FRK at the protein level. Interestingly, the stable overexpression of FRK in MDA-MB-231 cells altered the morphology of the cells from stellate to a more rounded phenotype (Figure 4C). Yim *et al*. described their MCF-7 cells as round and observed a dramatic morphological change to stellate-like in these cells upon the exogenous expression of FRK [8]. It is unclear why FRK would induce different morphological phenotypes in the MDA-MB-231 cell line, which we used in our study, and MCF-7 in the Yim *et al*. study [8]. Nonetheless, since stellate/FRK-negative MDA-MB-231 cell line is characterized as highly invasive [47] and we observed that overexpression of FRK alters the morphology to rounded, we hypothesized that the tumor suppressor activity of FRK might play a significant role in the inhibition of cell invasion. We found that the stable overexpression of FRK-WT or constitutively active FRK-Y497F led to a striking decrease in MDA-MB-231 proliferation, colony formation, migration, and invasion (Figures 4 and 5). However, while FRK-WT and FRK-Y497F had a similar effect on cell proliferation, the effect of FRK-Y497F was greater than FRK -WT in the inhibition of cell migration and invasion (Figure 5).

We next investigated the mechanism of action of FRK in our FRK-overexpressing MDA-MB-231 cell lines. Yim *et al*. previously demonstrated that in breast cancer cells, FRK acts by inhibiting the PI3K/Akt signaling via the phosphorylation and stabilization of the tumor suppressor PTEN [8]. A more recent study indicated that FRK associates with epidermal growth factor receptor (EGFR), induce its internationalization and thus inhibiting EGFR signaling [14]. Since the overexpression of constitutively active FRK-Y497F results in the phosphorylation of numerous targets (Figure 3C), it possible that the PI3K/AKT and EGFR pathways are not the sole cellular pathways regulated upon FRK activation. The tyrosine kinome is known to regulate various phospho-tyrosine signaling networks that mediate numerous biological processes including cell proliferation, migration, and invasion [48]. We, therefore, used a kinome-signaling array to identify potential FRK-regulated signaling pathways [30, 33]. These arrays have applied to understand mechanisms and identify biomarkers of some pathological states including cancer [49, 50]. We found that the presence of FRK resulted in the regulation of several signaling pathways including the downregulation of JAK/STAT, PI3K/Akt and MAPK signaling pathways (Figure 6A). The most significant effect of FRK was on the inactivation of STAT3 (JAK/STAT signaling) by both FRK-WT and FRK-Y497F (Figure 6A). Although, FRK-VK and VF mutants were shown to increase STAT3 phosphorylation in liver cancer [6]. However, when these mutants were transfected into breast cancer cell line, we observed no effect on STAT3 phosphorylation, although these mutants were constitutively active (Supplementary Figure 1A). It is possible that the function of these FRK mutants is tissue specific. We also observed a reduction in pAkt levels (PI3K/Akt signaling) and decrease in the levels of pMEK 1/2, p-p38 (MAPK signaling) and pJNK pathways were also inhibited in the presence of FRK (Figure 6A) In glioma cells, FRK was reported to reduce cell migration and invasion via inhibiting JNK activation [12]. Interestingly, we also found a decrease in pJNK in MDA-MB-231 stably expressing FRK-Y497F, upon activation of JNK pathway with anisomycin (50 ng/mL) at different time intervals. (Supplementary Figure 3). Although we saw a decrease in pMEK1/2 and an increase in the activation of ERK1/2 in the presence of FRK, the reasons for this opposing action of FRK are not entirely clear. However, a previous study has shown murine FRK-transgenic mice to demonstrate higher phosphorylation level of ERK1/2 but lower levels of phosphorylated p38 in islet cells compared with control islets [51]. Also, Jin *et al*., have shown that FRK phosphorylates EGFR at Y1173, hence leading to the internalization/degradation of EGFR [52], this could be the reason why there was a decrease in pMEK1/2 with the overexpression of FRK. Furthermore, phosphorylation of EGFR on Y1173 has been shown to phosphorylate ERK1/2 leading to its activation [52, 53]. It is possible that the increase in ERK1/2 phosphorylation is through FRK phosphorylation of EGFR at Y1173. Also, phosphorylation of ERK 1/2 has also been shown to negatively regulate the activation of STAT3 (Y705) in certain cells [54, 55]. Phosphorylation of STAT3 (S727) has been reported to negatively modulate STAT3 activation (phosphorylation at tyrosine 705) [56]. Indeed, we have shown that FRK enhances pSTAT3 (S727) (Supplementary Figure 4). It is worth mentioning we observed no change in the level of pSTAT3 with the knockdown of FRK in SKBR3 as well as MCF7 breast cancer cell lines. However, when we examined the phosphorylation status of pSTAT3 (S727) in SKBR3, we expected to see a decrease in pSTAT3 (S727) with the knockdown of FRK as there was an increase in pSTAT3 (S727) with overexpression of FRK. We, however, saw no change in the phosphorylation levels of pSTAT3 (S727) with the knockdown cells. This could be the reason why the knockdown of FRK had no significant effect on pSTAT3 (705) in both MCF-7 and SKBR3 breast cancer cell lines. Although, it is possible that we might see the effect if FRK was completely knockout from SKBR3 or MCF-7 breast cancer cell lines. We will further investigate this in future.

Because of the significant suppression of STAT3 activation by FRK (WT and Y497F), we checked if there was any correlation between FRK and pSTAT3 expression in a panel of 14 breast cancer cell lines. We found about 75% inverse correlation between FRK and pSTAT3 in the 14 breast cancer cell lines tested (Supplementary Figure 5), which aligns with our hypothesis that FRK is a negative regulator of STAT3 signaling. Several of STAT3 downstream target genes associated with tumorigenesis have been previously validated [39]. Some of the well-characterized STAT3 target genes include Survivin, matrix metalloproteinase 1 (MMP-1), cyclin D1, BCL2, cMYC and MCL1 [39]. Thus, we examined the effect of FRK on selected STAT3 target genes (Survivin, MMP-1 and cyclin D1). Survivin (encoded by BIRC5 gene) is an inhibitor of caspases, while MMP-1 promotes invasiveness via the degradation of the basal membrane and cyclin D1is a cell cycle regulator [39]. The presence of FRK-WT or FRK-Y497F reduced the mRNA expression of Survivin, and MMP-1, the mRNA expression of cyclin D1 was only reduced by FRK-WT (Figure 8A). However, the levels of Survivin and MMP-1 were upregulated in FRK knockdown SKBR3 and MCF-7 breast cancer cells (Figure 8B and 8C). The most dramatic effect of FRK was observed with Survivin. Survivin is a member of the inhibitor of apoptosis (IAP) family, and one of its functions is to inhibit caspase activation and thereby negatively regulate apoptosis [38]. Studies on invasive breast carcinoma demonstrated a correlation between STAT3 activation and Survivin expression [57]. In our future studies, we will examine how the expression of FRK correlates with STAT3 activation and Survivin expression. Although, the knockdown of FRK had no significant e ffect on STAT3 phosphorylation but had a significant effect on STAT3 target genes in both MCF-7 and SKBR3 it is possible that the downregulation of cyclin D1, MMP-1, and survivin by FRK is through other signaling pathways other than STAT3 signaling pathway. Based on this, we conclude that FRK might not regulate STAT3 signaling.

EMT is a mechanism that enhances metastasis of breast cancer by enabling epithelial cells to become more like mesenchymal cells with increased motility and invasiveness [58]. Mesenchymal markers include vimentin, fibronectin, slug, snail, and N-cadherin. E-cadherin is a classic epithelial marker. First, we found an inverse correlation between FRK and the mesenchymal marker, fibronectin in the Basal B breast cancer cell lines, MDA-MB-231, BT549 and Hs578T (Figure 9A). Vimentin, fibronectin, slug and N-cadherin were downregulated, while the expression of E-cadherin was upregulated with FRK overexpression in MDA-MB-231 cells (Figure 9E and 9F).

E-cadherin is a cell-cell adhesion molecule that is essential for the formation and maintenance of the epithelium [59]. Downregulation or loss E-cadherin expression or any other mechanisms that interfere with the integrity of the cell-cell interaction are phenomena in many cancers [60]. Cell-cell adhesion is altered by a switch from E-cadherin to N-cadherin expression (the so-called “cadherin switch”) [61]. The role of E-cadherin has been defined as anti-invasive or tumor suppressive because the loss of E-cadherin correlates with the loss of the epithelial morphology and in most cases with the acquisition of metastatic potential by the cancer cell [62]. In our study, we found that these properties are mirrored by FRK.

The trend of correlation of FRK with the epithelial marker, E-cadherin that we observed in our study was consistent with the mRNA expression dataset mined from the Affymetrix platform 133plus2. FRK transcript levels were positively correlated with the transcript levels of E-cadherin and Cytokeratin 18 as well as in the breast cancer tissues mined from TCGA database where FRK correlated positively with E-cadherin in both normal and breast tumor tissue samples. Although, as expected, a negative correlation was observed with the transcript levels of vimentin, N-cadherin, fibronectin, and TWIST in the breast cancer cells lines mined from GEO accession numbers GSE10021, GSE10843, GSE3156, GSE10890 and GSK's cell line project (https://array.nci.nih.gov/caarray/project/woost-00041/). We, however, did not see an inverse correlation between FRK and Fibronectin/Vimentin in the breast tumor samples. We believe that this may be due to the heterogeneous nature of the breast tumor sample cohort, represented by various breast cancer subtypes. The TNBC subtype, for instance, is sub-classified as, luminal androgen receptor positive, basal-like-1, basal-like-2, immunomodulatory, claudin-low-enriched mesenchymal, and mesenchymal stem-like (MSL) [63]. We noted that FRK expression was low/lost in the mesenchymal-like basal B subset of TNBCs. Therefore, the availability of a stratified mesenchymal-like subset of breast cancer patient samples in the TCGA database or any other database will be of help to further validate the correlation between FRK and the mesenchymal properties of breast tumor cells. Furthermore, the overexpression of FRK in both MDA-MB-231 and Hs578T lead to the decrease in menschenmyal markers such as fibronectin. Based on these findings, it is possible that the suppression of EMT by FRK is not only limited to MDA-MB-231 cells but affect all basal B cells.

Our results as a whole suggest that FRK either may play a role in the maintenance of the cell-cell interaction and the protection of the normal epithelium by upregulating E-cadherin and inhibiting EMT via the downregulation of Vimentin, fibronectin and N-cadherin. Therefore, the restraint of EMT might be one of the mechanisms underlying the anti-migration/invasion effect of FRK in breast cancer cells.

## MATERIALS AND METHODS

### Cell cultures

Breast cancer cell lines (AU565, BT20, MDA-MB-231, MDA-MB-468, HCC 70, BT 549, SKBR3, T47D, MCF 10A, MCF7) and human embryonic kidney 293 (HEK293) cells were obtained from the American Type Culture Collection (ATCC, Manassas, VA, USA). The cell lines were cultured in high glucose (4.5 g/l), Dulbecco's modified Eagle's medium (DMEM) supplemented with 10% bovine calf serum (Thermo scientific, Logan, USA) and contained 4mM L-glutamine, 100 units/ml penicillin, 100 mg/ml streptomycin (Sigma-Aldrich, St Louis, MO, USA).

### Antibodies and inhibitors

The following primary antibodies were purchased from Santa Cruz Biotechnology (California, USA): FRK (N19, sc-916), SLUG (sc-166476), Fibronectin (sc-8422) anti-GFP (sc-8334), β-actin (sc-130300), pTyr 20 (sc-508), pSTAT3- S7272 (sc-8001), JNK 1/2 (sc-137020), pJNK (sc-81502), p38 (sc-535), p-p38-Thr180/Tyr182 (sc-17852) and β-tubulin (sc-9104). STAT3, pSTAT3 705 (9145S), AKT (9272S), pAKT-S473 (4058S), MEK1/2 (9126) and pMEK1/2-S217/21 (9154S), were purchased from Cell Signaling (Massachusetts, USA). JNK activation was inhibited with 50ηg/mL anisomycin (Sigma-Aldrich, # A9789) for the indicated time-periods.

### Transient transfections and generation of stable cell lines

Transient transfection was done using HEK 293 cells. The cells were cultured in 6-well plates and were transiently transfected with a total of 2.5 μg of DNA using 1% polyethyleneimine ‘Max’ (PEI) (Polysciences Inc., Warrington, PA, USA). For each well, 2.5 μg of DNA was added to 107.5 μL of sterile 0.15M NaCl in a microcentrifuge tube and vortexed gently for 10s. 15 μl 0.1% PEI was added to the DNA mixture and vortexed gently for 10s. The DNA–PEI complex was incubated for 10 min at room temperature, and the mixture was added dropwise to wells containing 2 mL of complete media and incubated at 37°C. The cells were incubated for 24 h post transfection and harvested the next day. Transient knockdown of FRK in SKBR3 and MCF7 was carried using FRK-siRNA (shRNA) (Santa Cruz, CA, USA), as recommended by the manufacturers. MDA-MB-231 stably expressing FRK-Wild-type and FRK-Y497F was generated using the method previously described [28]. Pools of MDA-MB-231 cells stably expressing, LPC-FRK-WT and LPC-FRK-Y497F fusions were selected with puromycin (Sigma-Aldrich).

### Preparation of cell lysates

Cells were lysed using freshly prepared lysis buffer (20 mM Tris ph 7.5, 1% triton, 150 mm NaCl, protease inhibitors: Aprotinin 5 mg/l and PMSF 0.1 mM) and centrifuged at 14,000 rpm for 15 minutes at 4°C. Whole-cell lysates was obtained using SDS sample buffer (50 mM Tris/HCl (pH 6.8), 2% SDS, 0.1% Bromophenol Blue and 10% glycerol).

### Proliferation

Cell proliferation was assessed by measuring mitochondrial viability. A Cell Counting Kit-8 (CCK-8) (Dojindo, CK04-05) was used according to the manufacturer's protocol. Mitochondrial viability was measured by the reduction of 2-(2-methoxy-4-nitrophenyl)-3-(4-nitrophenyl)-5-(2,4-disulfophenyl)-2H-tetrazolium) by dehydrogenase to form an orange water-soluble formazan dye. Exponentially growing stable cells were seeded in 96-well plates (BD, 353077) and cultured in 100 μL culture medium (1000 cells/well). The cell proliferation assay was performed for 4 days (after 0, 24, 48, 72 and 96 hours). 10 μL CCK-8 solution was added to each well, and then the cells were incubated for 2 hours. Following treatment with CCK-8, absorbance at 485 nm was measured using a POLAR Star OPTIMA microplate re ader (BMG Labtech, 413-1040).

### Soft agar assay

0.61% (w/v) semisolid bottom agar containing 10% FBS culture medium was prepared and poured on 6 cm cell culture plates (Sigma-Aldrich, D8054). MEM Vitamin Solution (100×) was added to boost cell growth and viability. Stable cells were resuspended in 0.36% (w/v) top agar. Top and bottom agar contained the same components except for the percentage of agar. Low-melting top agar mix was then layered on the bottom agar. Plates were overlaid with fresh agar every 7 days to maintain the nutrient elements. Three parallel plates were set up for each cell line including the control. Colonies were counted after a 3-week incubation. Colonies larger than ~0.1 mm in diameter were considered as positive.

### Cell migration (wound-healing) assay

A confluent monolayer of cells was wounded by scratching a cross on a 10cm cell culture plate with a 1000 μL sterile pipette tip. The old culture medium was then aspirated and replaced with new culture medium supplemented with 10% FBS. The center of the wound was photographed by using an inverted microscope (Olympus, CK2) at time points of 0, 12, 18, 24 and 36 hours. The percentage of open area was analyzed and quantified by the TScratch software [29]. Each independent experiment was repeated at least three times.

### Invasion assay

Cell invasion assay was performed by using transwell plates incorporating a polycarbonate membrane with 8.0 μm size pore (Corning, 3422) following the modified method which was originally described by [28]. Inserts were pre-coated with 100 μL 0.15% gelatin and incubated at 37°C for 1 hour to produce a three-dimensional gel. Cells were counted and resuspended (1×10^5^ cells/well) in serum-free culture medium and added into the inserts. Each insert was placed in the lower chamber containing 10% FBS culture medium. Following 4 hours’ incubation, the culture medium in insert and chamber were removed, and non-migrated cells were swabbed by Q-tips from the upper surface of the insert. The membrane was fixed with 20% methanol for 30 minutes at room temperature. The cells that attached to the polycarbonate membrane at the bottom of the insert were stained with crystal violet and used to assess the degree of invasion. The experiments were performed in three replicates.

### Immunoblotting

Proteins derived from whole cell lysates were resolved via SDS-PAGE in 10% polyacrylamide gels. The resolved proteins were then transferred onto nitrocellulose membranes (Bio-RAD, Hercules, CA, USA) and immunoblotted with the appropriate antibodies via overnight incubation at 4°C. The membranes were washed three times with PBS and incubated for 1 h with fluorescent secondary antibodies (LI-COR Biotechnology, Guelph, ON, Canada). LI-COR Odyssey imaging system (LI-COR Biotechnology) was used to obtain the protein images.

### Kinome assay

Kinome assay was performed according to Jalal *et al.* [30]. HEK 293 cells transfected with FRK-WT and untransfected HEK293 as control were both cultured to confluency in 10 cm culture plates respectively.Briefly, cell pellets were lysed with 100 μL lysis buffer (20 mM Tris-HCl pH 7.5, 150 mM NaCl, 1 mM EDTA, 1 mM EGTA, 1% TRITON, 2.5 mM sodium pyrophosphate, 1 mM Na3 VO4, 1 mM NaF, 1 μg/mL leupeptin, 1 g/mL aprotinin, 1 mM PMSF), incubated on ice for 10 min and then spun in a microcentrifuge for 10 min at 4°C. A 70-μL aliquot of this supernatant was mixed with 10 μL of activation mix (50% glycerol, 50 uM ATP, 60 mM MgCl2, 0.05% v/v Brij-35, 0.25 mg/mL BSA, 2 mCi/mL γ-32P-ATP) and incubated on the array for 2hr at 37°C. Finally, slides were washed once with phosphate saline (PBS) (1 × solution; pH 7.3) containing 1% TRITON® X-100, twice with 2M NaCl containing 1% TRITON® X-100 and finally in demineralized H_2_O. Following air drying, arrays were exposed to a phosphoimager screen for one week. Images were obtained by scanning the screen with TYPHOON® scanner (GE Healthcare) and then loaded on ARRAYVISION® (Image Research). Intensity values for the spots and background were obtained and normalized. Statistical analyses were performed with GENESPRING® (Agilent Technologies) software.

### Real-Time PCR: RNA isolation, reverse transcription, PCR and real-time PCR

Total RNA was isolated from cell lines using RNeasy Plus Mini Kit (Qiagen, Mississauga ON). The RNA quantity and quality were analyzed using a spectrophotometer and gel electrophoresis, 1.0 μg of total RNA was used as a template to generate cDNA using the Iscript cDNA Synthesis Kit (Bio-Rad, United States). The cDNA synthesized was used as a template in quantitative RT-PCR reactions. Quantification of the expression *GAPDH*, *SURVIVIN*, *MMP1*, *FN1*, and *VIMENTIN* was performed using primers listed in the Supplementary Table 1 and sybr green SsoFast™ EvaGreen Supermix^(R)^ (BIO-RAD) as described previously [31]. The expression of *FRK*, *SLUG*, and *GAPDH* was determined using TaqMan probes Hs00176619_m1, Hs00950344-m1, and Hs02758991-g1, respectively as recommended by the manufacturer (Life Technologies, Burlington, ON, Canada). Briefly, to each well, 0.5 μL of probes for the target and housekeeping genes were added to the cDNA (0.6 μL) and TaqMan^(R)^ Master Mix (5 μL) (Life Technologies, Burlington, ON, Canada), then topped up with dH_2_O to a volume of 10 μL. Probes for *GAPDH* and target genes (*FRK* and *SLUG*) were labeled with VIC™ and FAM™ dyes, respectively. The expression of both the target and housekeeping genes were done within the same well and detected using an Applied Biosystems™, Step One Plus qRT-PCR machine (Life Technologies, Burlington, ON, Canada).

### Gene expression datasets *(in-silico)*

The gene expression profiles of the different breast cancer cell lines were first analyzed using a bioinformatics software GENT “gene expression across normal and tumor” (http://medicalgenome.kribb.re.kr/GENT/reference.php). GENT utilizes datasets created by the Affymetrix platforms (U133A and U133plus2). The normalized expression profiles of target genes in all cancer cells generated from the Affymetrix platform 133plus2 was downloaded. The expression data of target genes in breast cancer cell lines were selected, cell line nomenclature was harmonized, and cell classified by their phenotype and molecular subtype (Basal A, BA; Basal B, BB; or Luminal, LU, Supplementary Table 5). Correlation analysis were run on all the breast cancer cells (n = 56) with GEO accession numbers GSE10021, GSE10843, GSE3156, GSE10890 and GSK's cell line project (https://array.nci.nih.gov/caarray/project/woost-00041/) to determine consistency across the different profiles.

### Tumor expression data

Expression datasets for breast cancer were downloaded from the online database, The Cancer Genome Atlas (TCGA; http://tcga-data.nci.nih.gov). There were 1104 breast tumors and 114 normal mammary tissue samples. We used the immunohistochemistry (IHC) data available for these samples, classified into three sub-types. Gene expression data normalized using RSEM (RNA Seq Expectation Maximization) algorithm was used to analyze the correlation in expression. The sub-type classification was analyzed using python scripts pylab. The scipy stat library was used to generate the graphs and Spearman rank correlation between FRK and EMT/MET markers.

### Statistical analysis

For statistical analysis, one-way and two-way analysis of variance followed by a post hoc Newman–Keuls test was used for multiple comparisons using GraphPad Software, San Diego, California, USA, www.graphpad.com. The results are given as the means ± s.d., nX3 unless otherwise stated. P≤0.05 was considered statistically significant. The gene expression data was analyzed by a one-way analysis of variance (ANOVA; SigmaStat Version 2.0, Jadel Corporation, San Rafael, CA, USA). Multiple range comparisons of paired means were done using a Fishers LSD test or the Newman-Keuls test. Level of significance was set at *P*<0.05. Data was reported as mean ± SEM. Pearson's correlations were done to evaluate the consistency of the data and the relationship between gene expression profiles in the different cell lines.

## CONCLUSIONS

**Figure 11:**
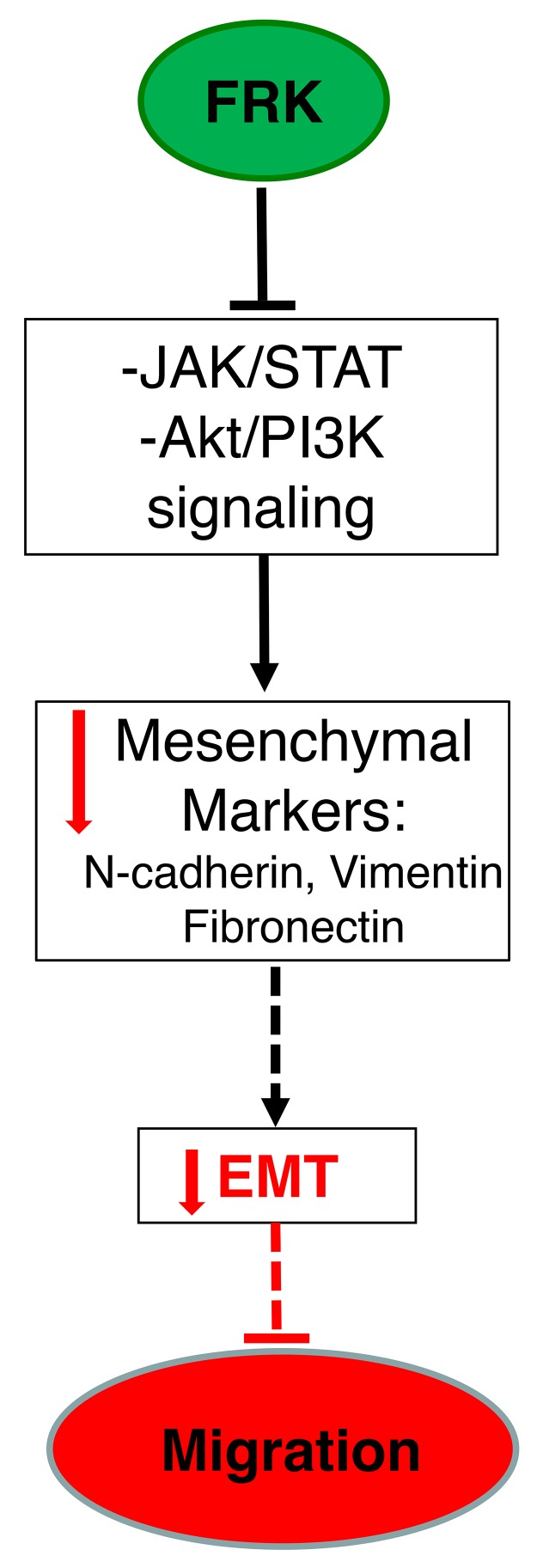
The summarized schematic depiction of the various potential mechanisms of FRK-mediated suppression of EMT in breast cancer cells Analyses of our peptide-array data revealed that the overexpression of FRK alters the activation status of key signaling proteins. These include AKT/PI3K and JAK/STAT3 signaling pathways. We validated that the overexpression of FRK in breast cancer cells suppresses STAT3 activation which leads to the suppression of mesenchymal genes: namely, N-cadherin, Vimentin and Fibronectin. Our data indicate potential mechanism by which FRK suppresses breast cancer cell migration is by the inhibition of epithelial-to-mesenchymal transition (EMT).

FRK is a non-receptor tyrosine kinase with unpredicted tumor suppressor activity. We have shown that stable overexpression of FRK suppressed cell proliferation, migration, invasion and colony formation. However, the data presented provide the first evidence that: 1) FRK is lost in breast cancer cells with mesenchymal phenotype ; 2) FRK expression correlates positively with epithelial markers and 3) the overexpression of FRK suppresses STAT3 activation leading to reduced STAT3 phosphorylation. Taken together, our findings indicate that inhibits breast cancer cell migration, potentially via suppression of EMT (Figure 11).

## DECLARATIONS

### Ethical approval and consent to participate

Not applicable

### Consent for publication

Not applicable

### Availability of supporting data

Not applicable

## SUPPLEMENTARY MATERIALS FIGURES AND TABLES



## Abbreviations

ATCC: American Type Culture Collection
ATP: adenosine triphosphate
BCL2: B-cell lymphoma 2
BD: Boyden
BFK: breast family kinase
BRCA1: breast cancer anti-estrogen resistance protein 1
CBCF: Canadian Breast Cancer Foundation
CCK-8: cell counting kit-8 () (Dojindo, CK04-05)
CCLE: cancer cell line encyclopedia
cDNA: complementary DNA
CIHR: Canadian Institutes of Health Research
DMEM: Dulbecco's modified Eagle's medium
EGFR: epidermal growth factor receptor
EMT: epithelial-mesenchymal transition
ERK: extracellular signal-regulated kinases
ETS: E-twenty-six transformation-specific
FBS: Fetal Bovine serum
FRK: Fyn-related kinase
FRK K262M: disruption of ATP binding; kinase-defective or Kinase-dead
FRK V378–F379 del (VF): deletion of amino acid on FRK kinase domain from valine 378-lysine 380 and insertion of glutamine
siRNA: short or small interfering RNA
shRNA: short or small hairpin RNA
WT: wild-type
FRK-Y497F: mutation of FRK regulatory tyrosine 497 to phenylalanine
GAPDH: glyceraldehyde 3-phosphate dehydrogenase
GRB: growth factor receptor-bound protein
HEK 293: human embryonic kidney cells 293
HER2: human epidermal growth factor receptor 2
IAP: inhibitor of apoptosis family
IHC: immunohistochemistry
IL-6: Interleukin 6
JAK: Janus kinase
JNK: c-Jun N-terminal kinases
LOH: loss of heterozygosity
LPC: lysophosphatidylcholine
MAPK: mitogen-activated protein kinase
MCL1: myeloid leukemia cell
MEK1/2: mitogen-activated protein kinase kinase
MEM: minimum essential medium
MET: mesenchymal-epithelial transition
MMP: matrix metalloproteinase 1
mRNA: messenger RNA
MSL: mesenchymal stem-like
MTT: -3-(4,5-dimethylthiazol-2-yl)- 2,5-diphenyl-2H-tetrazolium bromide
PBS: phosphate buffer / phosphate saline
PEI: polyethyleneimine
PI3K: phosphatidylinositide 3-kinases
PMSF: phenylmethylsulfonyl fluoride
PTEN: phosphatase and tensin homolog
PTK5: protein tyrosine kinase 5
pY20: anti-phosphotyrosine antibody
RSEM: RNA seq expectation maximization
RT-PCR: reverse transcription polymerase chain reaction
SCA: Saskatchewan Cancer Agency
SDS: sodium dodecyl sulfate
SDS-PAGE: sodium dodecyl sulfate-polyacrylamide gel electrophoresis
SFKs: Src-family kinases
SH2: Src homolog
2SH3: src homolog 3
SRMS: Src-related kinase lacking C-terminal regulatory tyrosine and N-terminal myristoylation sites
STAT: signal transducer and activator of transcription
TCGA: The Cancer Genome Atlas
TMA: tissue microarray
TNBC: triple-negative breast cancer
TNM: classification of malignant Tumors (T- size of tumor; N- lymph nodes; M- metastasis)
