# Supplementary material for: FRK inhibits breast cancer cell migration and invasion by suppressing epithelial-mesenchymal transition
Source: Oncotarget. 2017 Dec 6;8(68):113034–65. doi: 10.18632/oncotarget.22958 (PMC5762571; doi:10.18632/oncotarget.22958)
Supplement: Supplementary file 1 [file oncotarget-08-113034-s001.pdf]

FRK inhibits breast cancer cell migration and invasion by suppressing epithelial-mesenchymal transition

SUPPLEMENTARY MATERIALS

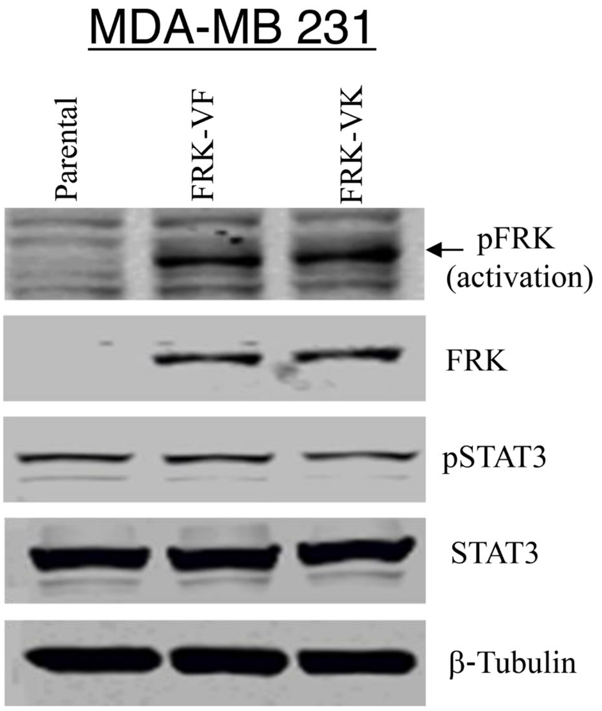

Supplementary Figure 1: Enzymatic activity of FRK variants, FRK V378–F379 del (VF), V378–K380 delinsE (VK) A. MDA-MB-231 cells were transiently transfected with GFP-tagged FRK VF, and GFP-tagged FRK-VK. Cells were lysed, the lysates resolved via SDS-PAGE and used in western blotting analyses. An anti -phosphotyrosine antibody, 4G10 was used to determine the tyrosine kinase activity. It determines the total tyrosine-phosphorylated proteins in the samples. Anti-FRK, pSTAT3, STAT3 and beta-tubulin antibodies were used to determine the expression of the transfected FRK variants.

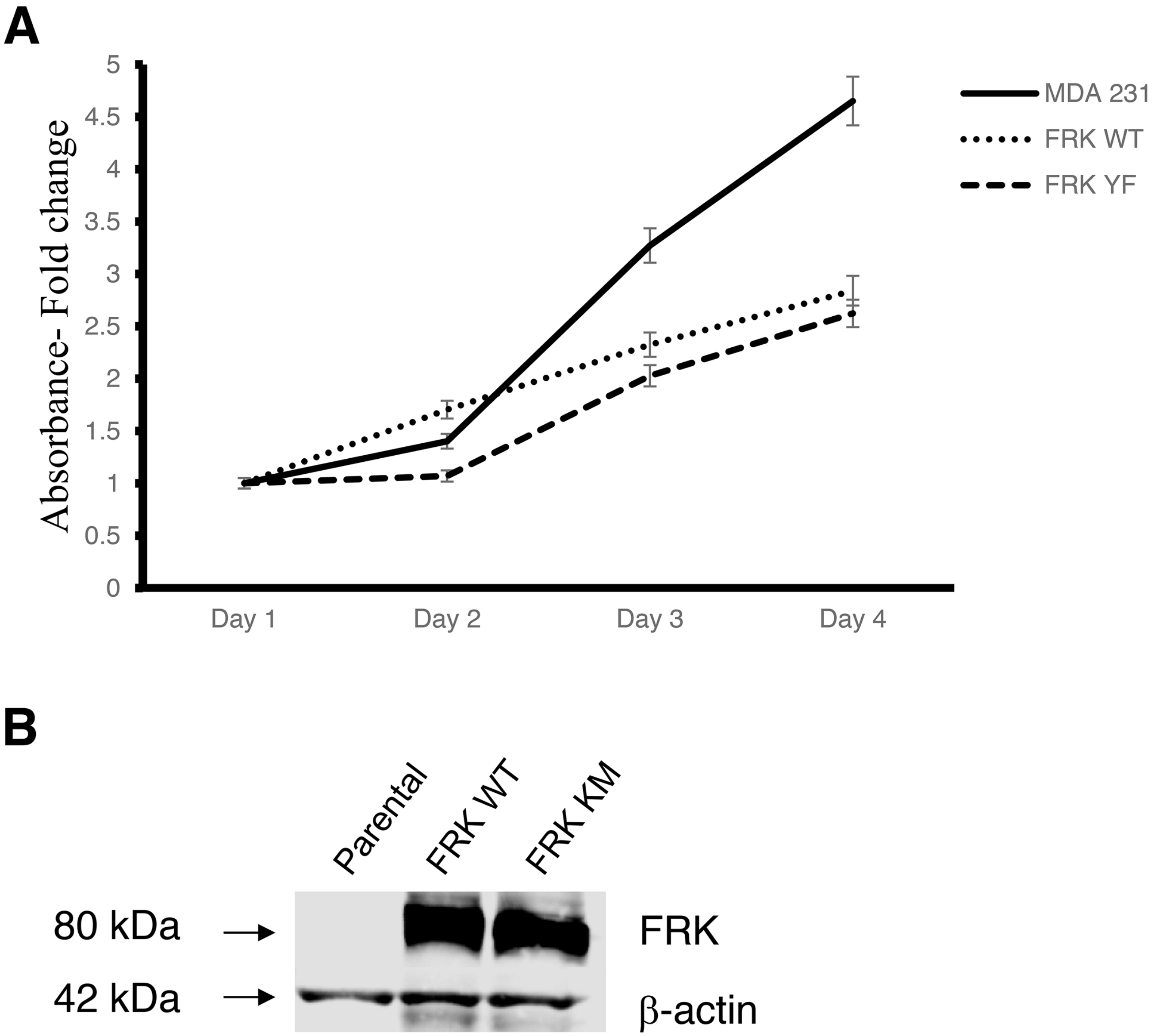

Supplementary Figure 2: Effect of FRK overexpression on cell proliferation. **(A)** Vectors encoding wild-type FRK (FRK-WT) or FRK Y497F (FRK-YF) were retrovirally introduced into MDA-MB-231 cells and polyclonal populations derived and designated as indicated. The empty vector-transduced cell line served as control. **(B)** Parental MDA-MB 231 and MDA-MB 231 cells transiently expressing FRK WT and FRK-KM (kinase-defective/dead mutant). Cells were lysed, the lysates resolved via SDS-PAGE and used in western blotting analyses. The protein expression of FRK was determined, βeta-actin served as the loading control. (*Continued*)

C

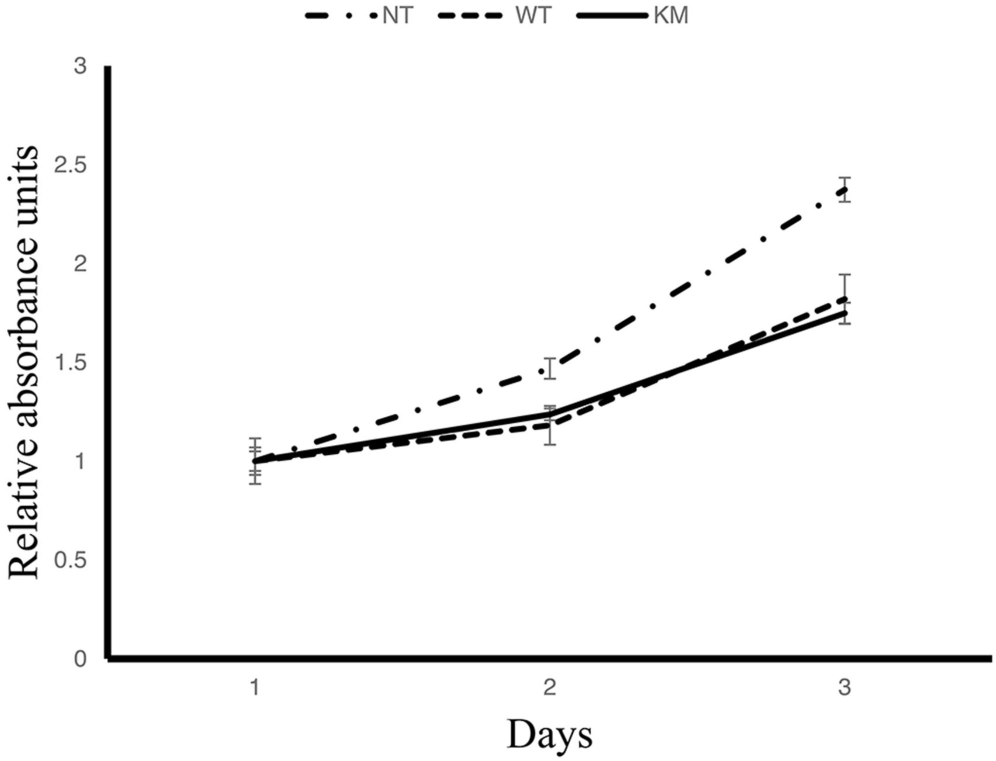

D

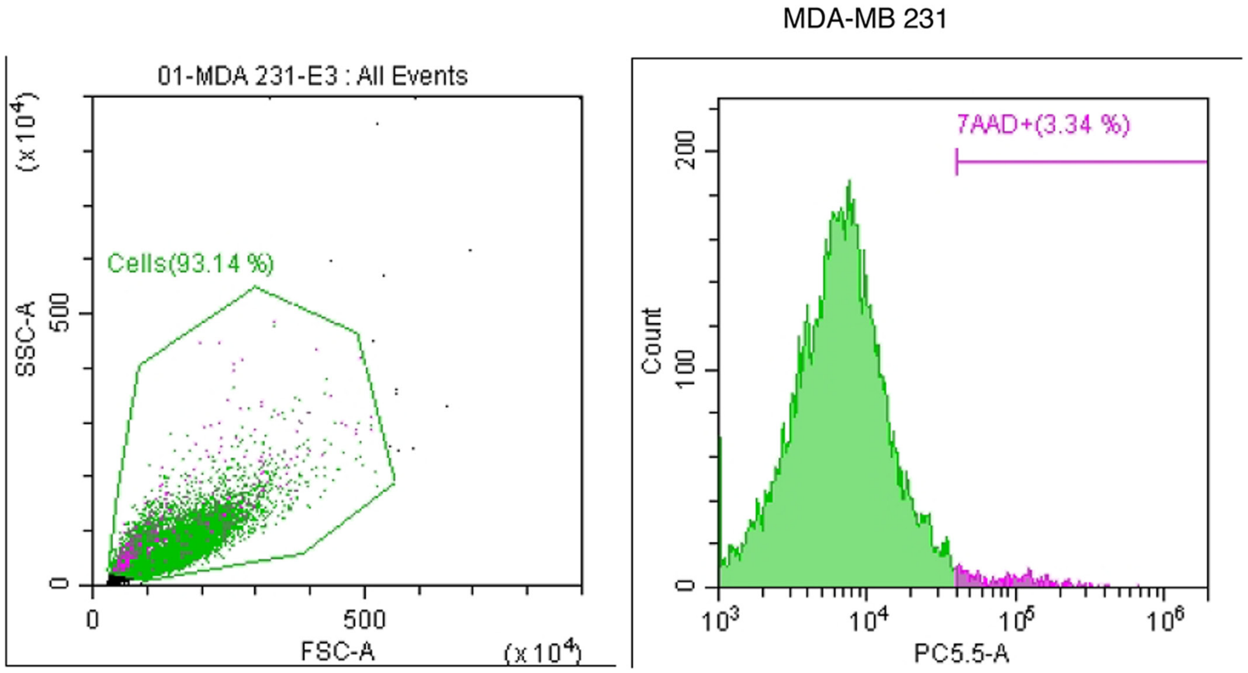

Supplementary Figure 2: (*Continued*) Effect of FRK overexpression on cell proliferation. **(C)** Cell proliferation rates of the indicated FRK transfected cell lines and the control cells were measured using the CCK8 assay. **(D, E and F)** The number of live and dead cells were quantified with a flow cytometer in the transiently transfected MDA-MB-231 cell lines using 7AAD assay. (*Continued*)

E

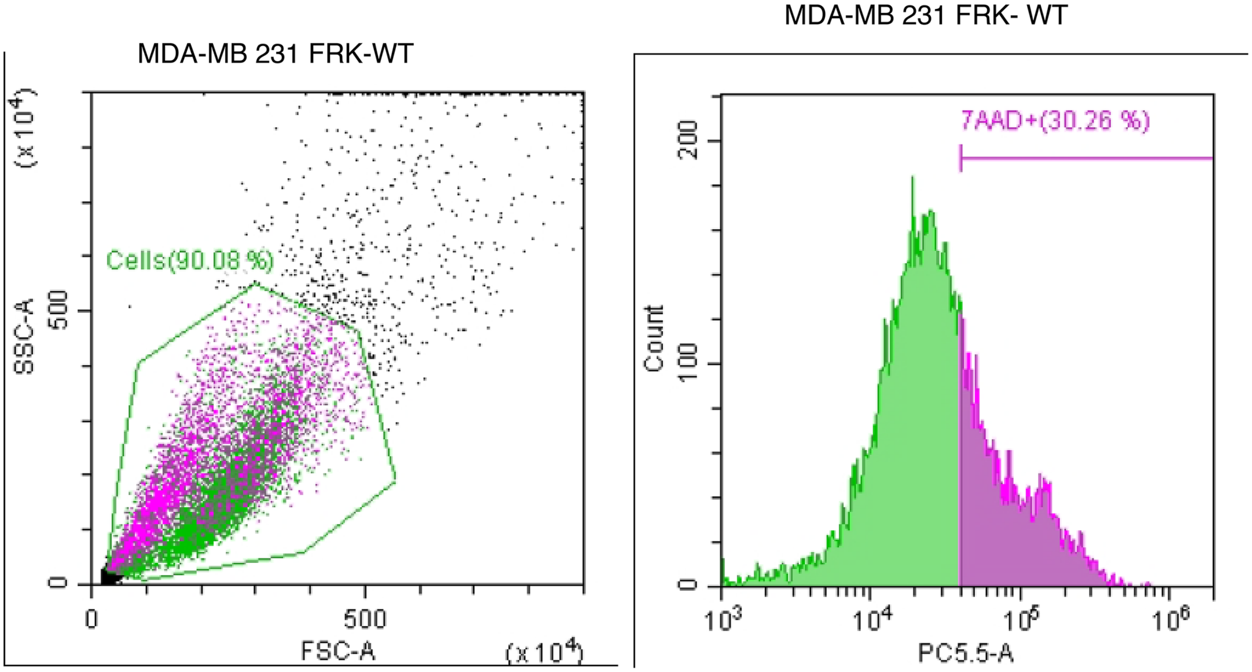

F

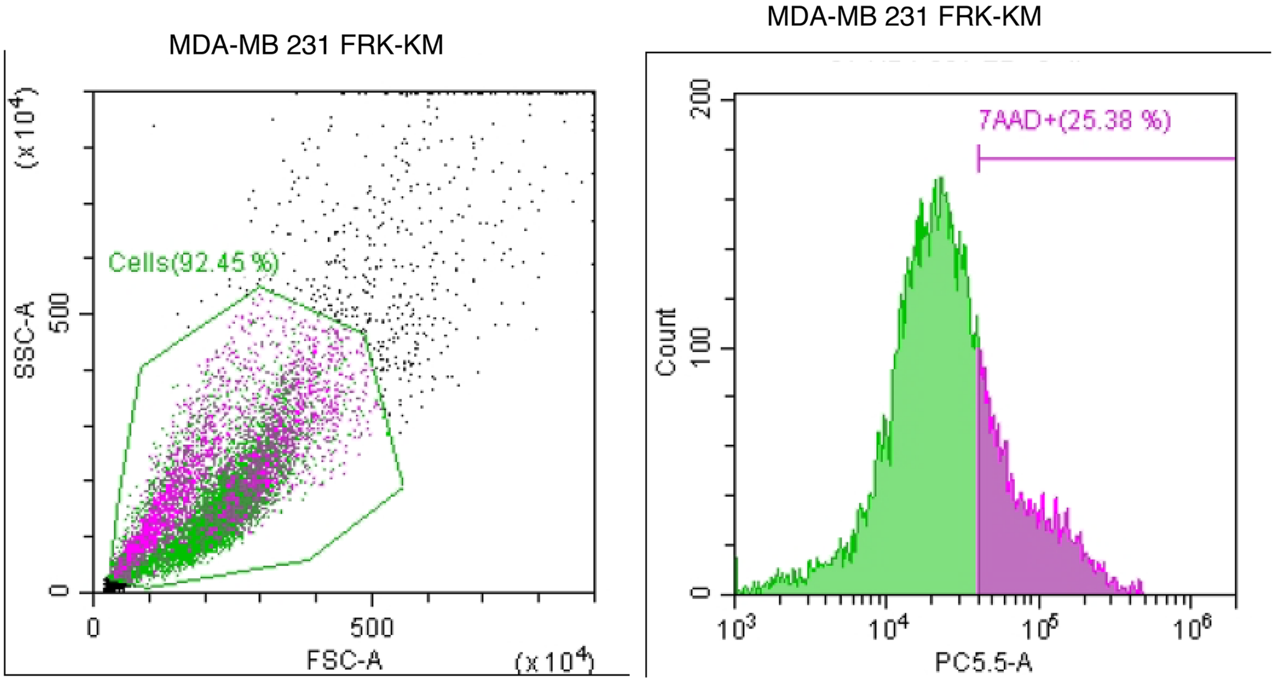

Supplementary Figure 2: (*Continued*) Effect of FRK overexpression on cell proliferation. E and F The number of live and dead cells were quantified with a flow cytometer in the transiently transfected MDA-MB-231 cell lines using 7AAD assay. (*Continued*)

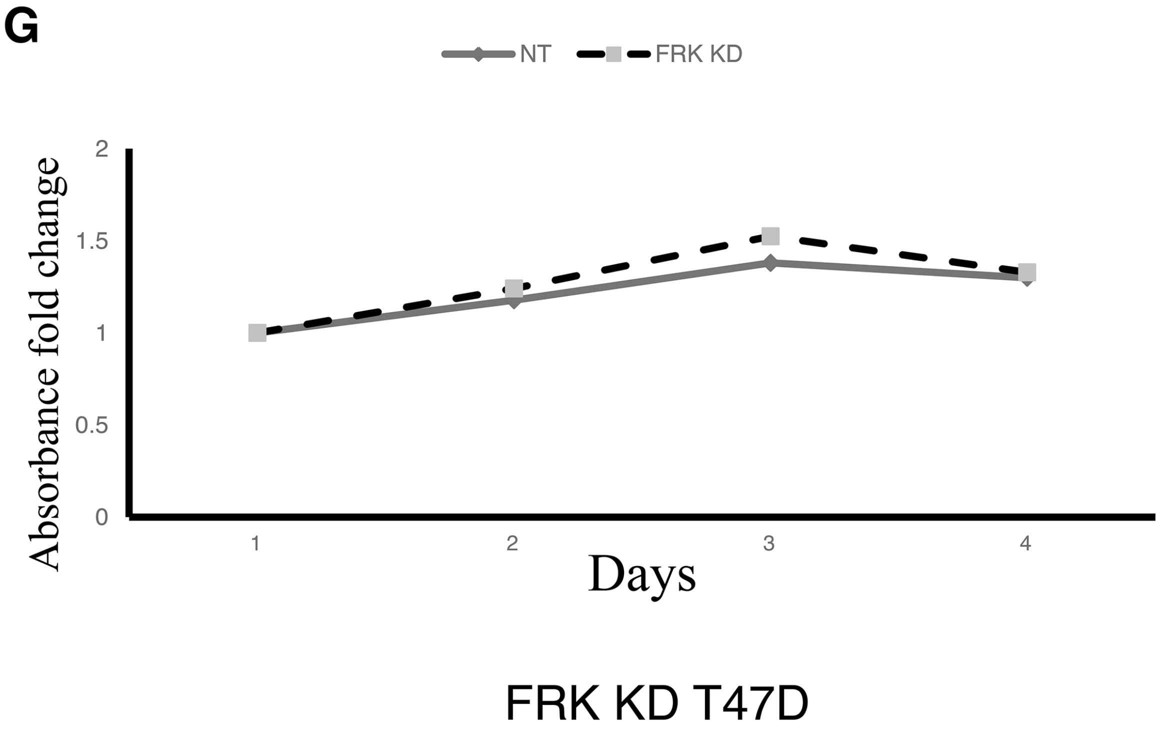

Supplementary Figure 2: (*Continued*) Effect of FRK overexpression on cell proliferation. (G) FRK was transiently knockdown in T47D, and cell proliferation rates were measured using CCK8 assay.

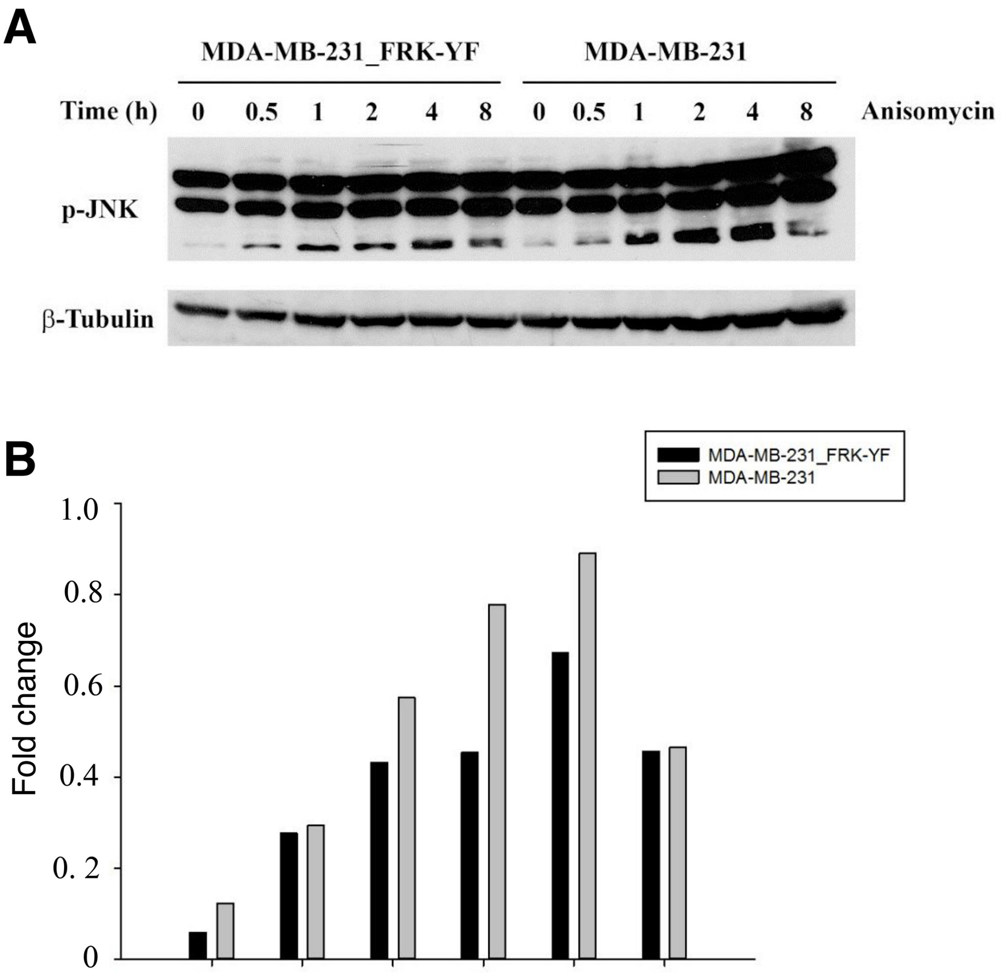

Supplementary Figure 3: Effect of FRK expression on the activation of JNK in anisomycin-treated MDA-MB-231 cells. **(A)** Parental MDA-MB-231 and MDA-MB-231 cells stably expressing FRK-Y497F (FRK-YF) were treated with 50ηg/mL anisomycin for the indicated time-periods. Cells at the indicated time-points were then lysed, and lysates probed for the expression of phospho-JNK. βeta-tubulin served as the loading control. **(B)** The expression levels of phospho-JNK from the immunoblotting analyses were quantified using the Image J software (Ver. 1.48).

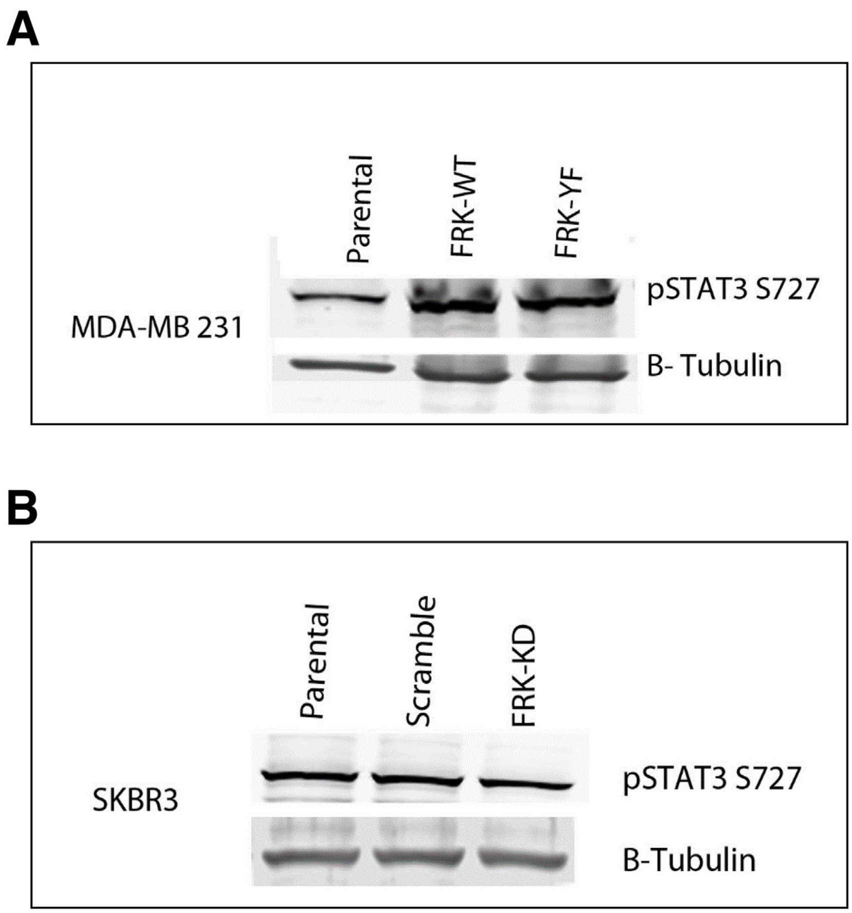

Supplementary Figure 4: Modulation of FRK expression and phosphorylation of STAT3 (S727) in breast cancer cells. Phospho-STAT3 (S727) levels were assessed via Western blotting using lysates corresponding to **(A)** Parental MDA-MB 231 cells, MDA-MB 231 cells stably expressing either FRK-WT or FRK-YF and **(B)** Parental SKBR3 cell, scramble siRNA-transfected or FRK siRNA-transfected SKBR3 cells. β-tubulin served as the loading control.

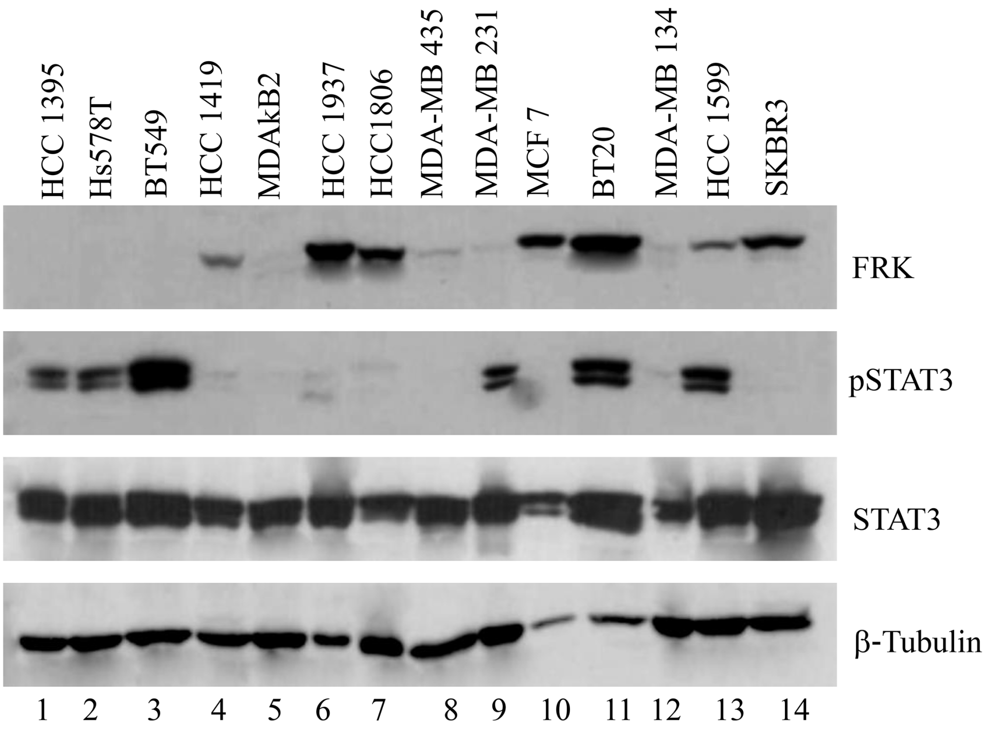

Supplementary Figure 5: FRK and pSTAT3 expression in several breast cancer cell lines. 14 breast cancer cell lines were probed for FRK, pSTAT3, STAT3 expression. β-tubulin was used as the loading control. **(A)** The cells were lysed, and western blotting analyses were done. Anti-FRK, pSTAT3, STAT3 and beta-tubulin antibodies were used to determine the expression of the different proteins

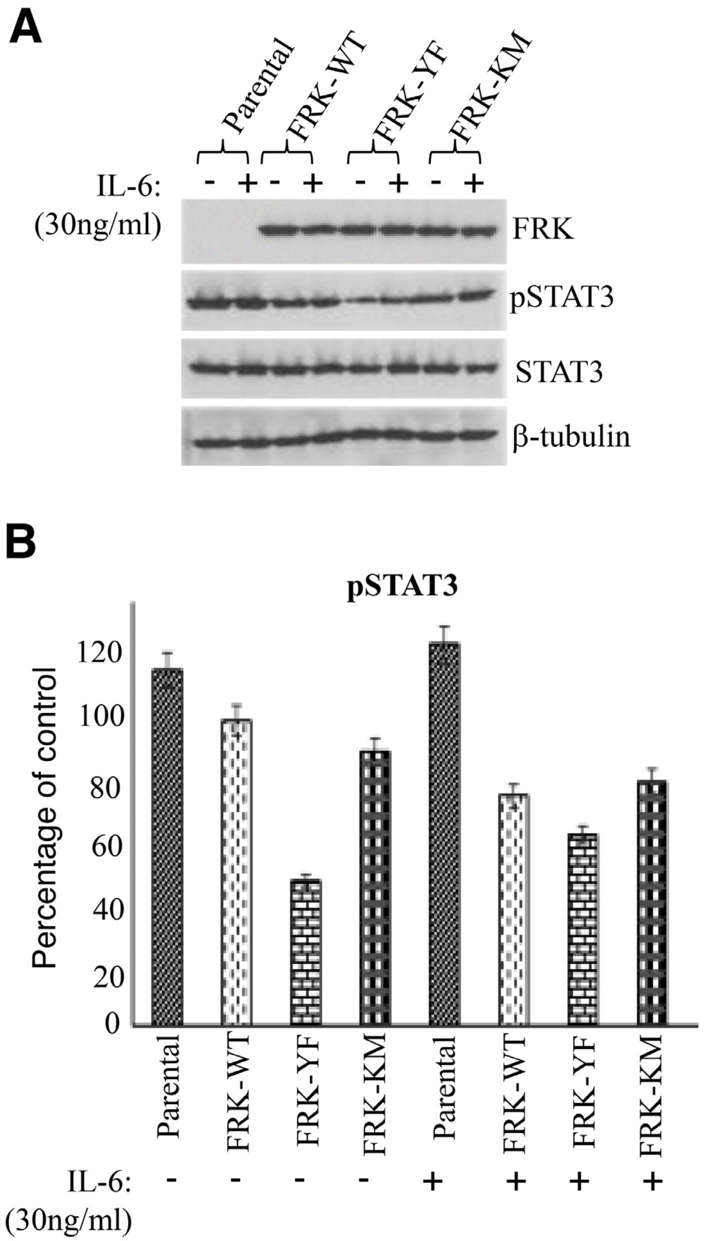

Supplementary Figure 6: Effect of FRK on IL-6-stimulated activation of STAT3. (A) Parental MDA-MB-231 cells or MDA-MB-231 cells transiently transfected with wild-type FRK (FRK-WT), kinase-dead FRK (FRK-K262M) or FRK-Y497F (FRK-YF) were stimulated with 30ηg/mL IL-6 or vehicle. Cells were harvested, lysed and probed for the expression of total STAT3 and phospho-STAT3 (Y705) by Western blotting using the appropriate antibodies. β-Tubulin was used as the loading control. **(B)** The expression levels of phospho-STAT3 from the immunoblotting analyses were quantified using the Image J software (Ver. 1.48).

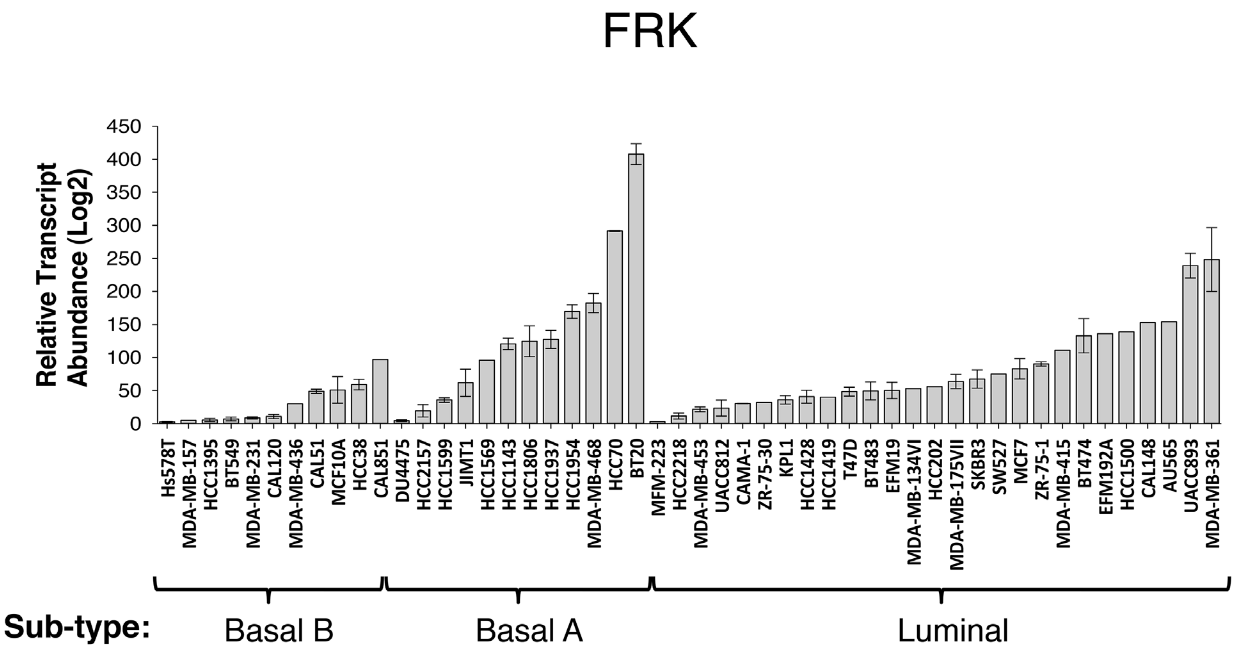

Supplementary Figure 7: Gene expression profile of FRK in Basal A, Basal B, and luminal breast cancer cell lines. FRK expression data corresponding to 56 breast cancer cells lines mined from GEO accession numbers GSE10021, GSE10843, GSE3156, GSE10890 and GSK's cell line project (https://array.nci.nih.gov/caarray/project/woost-00041/). The normalized expression profiles of target genes in all cancer cells generated from the Affymetrix platform 133plus2 was used.Breast cancer cell lines (n=56) were classified either as Basal A, Basal B or Luminal.

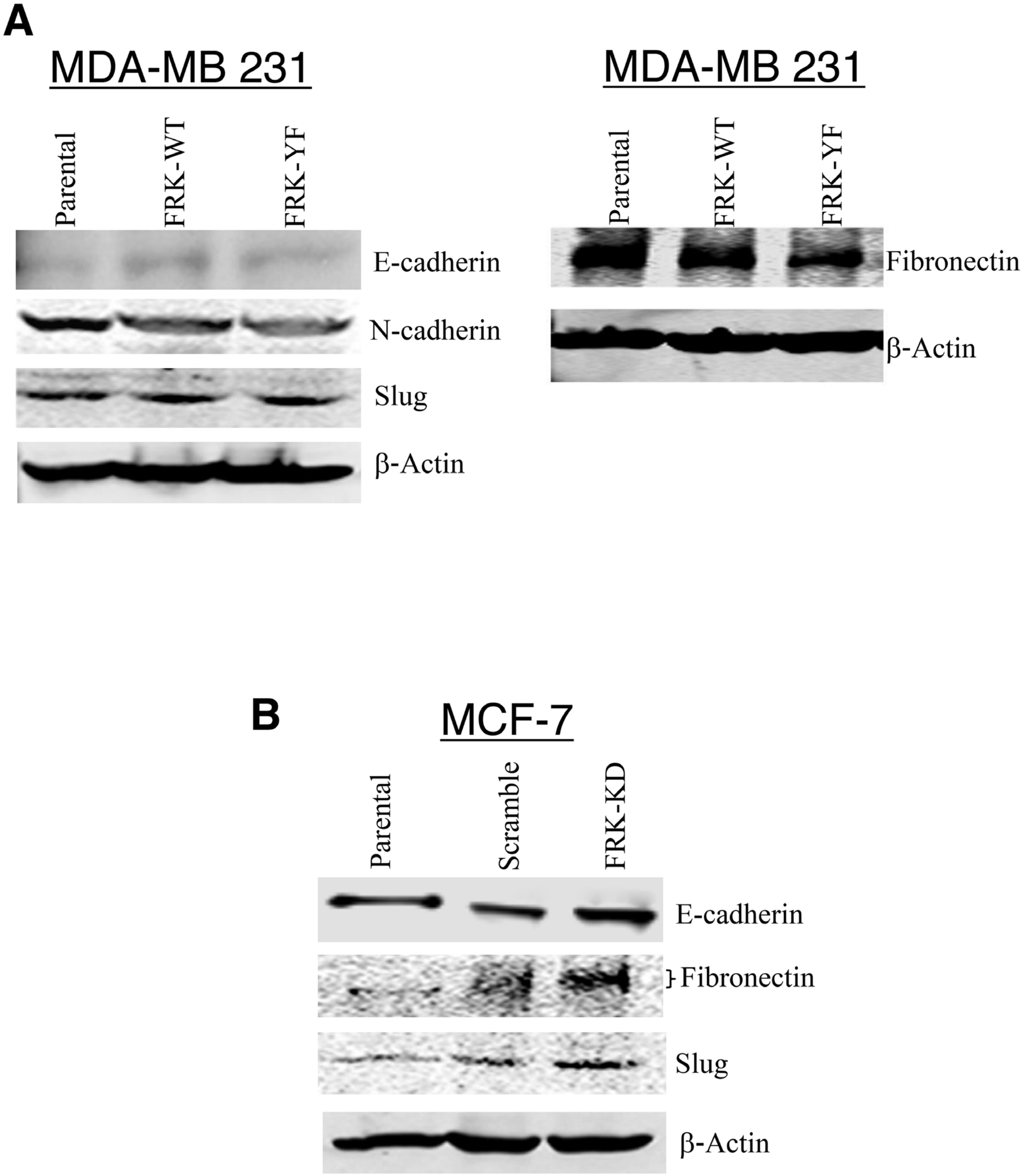

Supplementary Figure 8: Expression of epithelial and mesenchymal markers upon FRK-overexpression or FRK knockdown in breast cancer cells. (A) Lysates from parental MDA-MB-231 or MDA-MB-231 cells stably expressing either wild-type FRK (FRK-WT) or FRK-Y497F (FRK-YF) were probed for the expression of E-cadherin, N-Cadherin, and Slug (left panel), or Fibronectin (right panel) using appropriate antibodies. β-actin was used as the loading control. **(B)** Expression of E-cadherin, Fibronectin, and Slug was examined in MCF7 cells following the transient knockdown of FRK. β-actin was used as the loading control.(*Continued*)

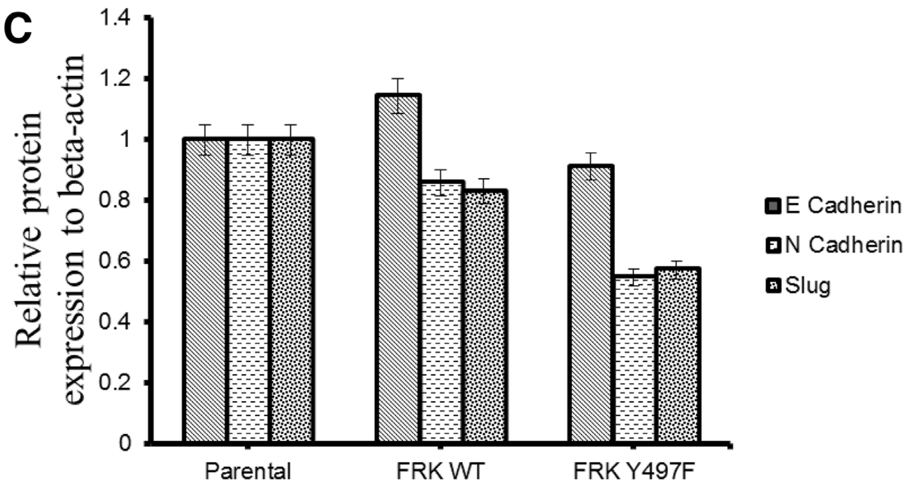

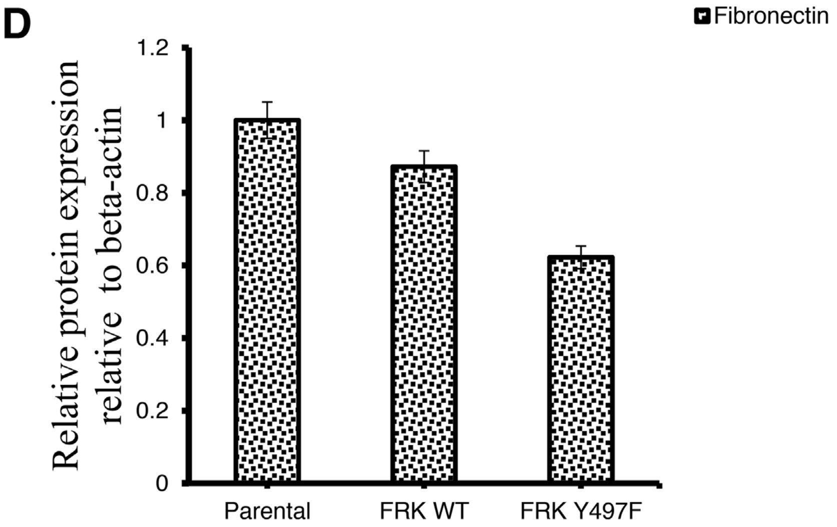

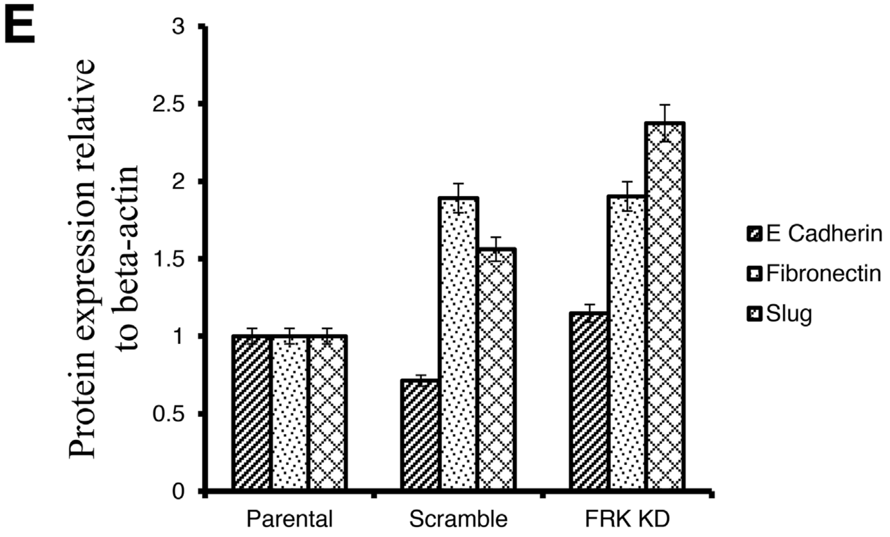

Supplementary Figure 8: (Continued) Expression of epithelial and mesenchymal markers upon FRK-overexpression or FRK knockdown in breast cancer cells. (**C, D and F**) The expression levels of indicated proteins from the immunoblotting analyses were quantified using the Image J software (Ver. 1.48).

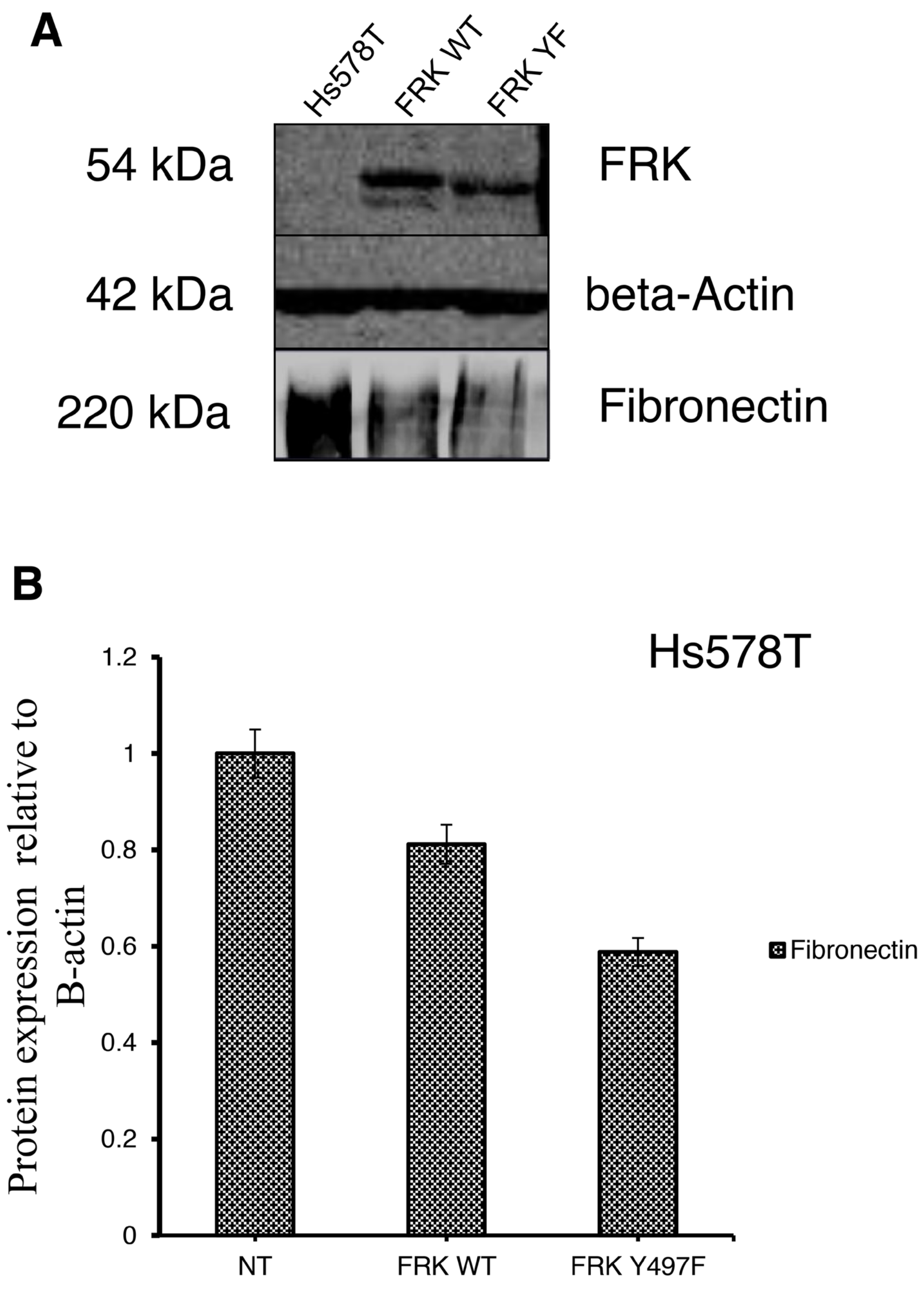

Supplementary Figure 9: Expression of mesenchymal marker in Hs578T overexpressing FRK. **(A)** Parental Hs578T and Hs578T cells transiently expressing FRK- WT and FRK-Y497F (FRK YF) were probed for the expression of Fibronectin using appropriate antibodies. β-actin was used as the loading control. **(B)** The expression levels of fibronectin from the immunoblotting analyses were quantified using the Image J software (Ver. 1.48).

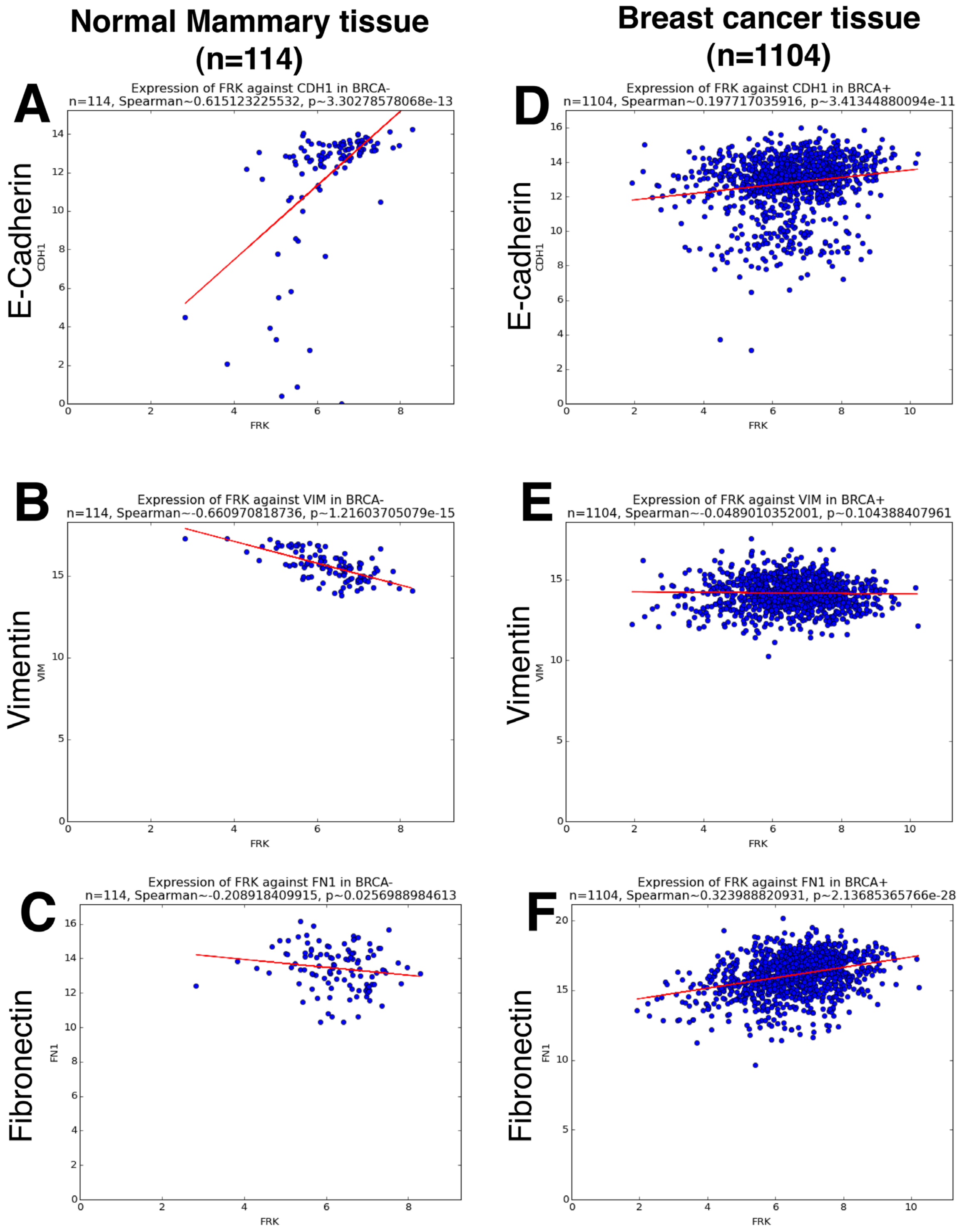

Supplementary Figure 10: FRK gene expression correlation with EMT markers. Gene expression from RNA Seq V2 data from 114 normal mammary tissues **(A, B, C)** and 1104 breast cancer samples **(E, F, G)** from TCGA were downloaded and analyzed for FRK, E-cadherin, Vimentin, and Fibronectin expression. The scatterplots represent correspondence of FRK expression with E-Cadherin (A and **D**), Vimentin (B and E) and Fibronectin (C and F) in TCGA Expression values are presented are log2 intensities. Included in the scatterplots are the Spearman rank correlation with significant P-value and lines showing the linear fit.

Supplementary Table 1: Primers used in the quantitative PCR analysis of transcript expression of the target genes relative to the house keeping gene (GAPDH) in breast cancer cells. Direction of the primers are as listed (forward or reverse).

Target Gene	Direction	Sequence
GAPDH	Forward	5′GTCAGTGGTGGACCTGACCT 3′
	Reverse	5′TGCTGTAGCCAAATTCGTTG 3′
SURVIVIN	Forward	5′GGACCACCGCATCTCTACAT 3′
	Reverse	5′GACAGAAAGGAAAGCGCAAC 3′
MMP1	Forward	5′GGACCACCGCATCTCTACAT 3′
	Reverse	5′GACAGAAAGGAAAGCGCAAC 3′
FIRBRONECTIN	Forward	5′CCCAACTGGCATTGACTTTT 3′
	Reverse	5′ CTCGAGGTCTCCCACTGAAG 3′
VIMENTIN	Forward	5′ GAGAACTTTGCCGTTGAAGC 3′
	Reverse	5′ TCCAGCAGCTTCCTGTAGGT 3′
CYCLIN D1 (CCND1)	Forward	5′ GATCAAGTGTGACCCGGACT 3′
	Reverse	5′ TCCTCCTCTTCCTCCTCCTC 3′

Supplementary Table 2: FRK expression and localization in a 6-cases/24-core breast cancer tissue array with matched adjacent normal breast tissue. The array includes patient clinical information such as TNM, stage and grade (BR243h, USBIOMAX)

Array position	Patient	Sex	Age	Pathology diagnosis	Grade	Stage	TNM	Type
A1	1	F	35	IDC	2	IIa	T2N0M0	Malignant
A2	2	F	35	IDC	2	IIa	T2N0M0	Malignant
A3	3	F	35	AN	–	–	–	NA
A4	4	F	35	AN	–	–	–	NA
A5	5	F	50	IDC	2	IIIa	T2N2M0	Malignant
A6	6	F	50	IDC	2	IIIa	T2N2M0	Malignant
A7	7	F	50	AT (DE)	–	–	–	NA
A8	8	F	50	AN (FF)	–	–	–	NA
B1	9	M	56	IDC	2	IIIa	T2N2M0	Malignant
B2	10	M	56	IDC	2	IIIa	T2N2M0	Malignant
B3	11	M	56	AN	–	–	–	NA
B4	12	M	56	AT (DE)	–	–	–	NA
B5	13	F	41	IDC	2	–	–	Malignant
B6	14	F	41	IDC (n) necrosis	2	–	–	Malignant
B7	15	F	41	AN	–	–	–	NA
B8	16	F	41	AN	–	–	–	NA
C1	17	F	30	IDC	2	IIb	T2N1M0	Malignant
C2	18	F	30	IDC	2	IIb	T2N1M0	Malignant
C3	19	F	30	AN	–	–	–	NA
C4	20	F	30	AN	–	–	–	NA
C5	21	F	40	IDC	2	IIb	T2N1M0	Malignant
C6	22	F	40	IDC	2	IIb	T2N1M0	Malignant
C7	23	F	40	AT (DE)	–	–	–	NA
C8	24	F	40	AN	–	–	–	NA

AN = Cancer adjacent normal breast tissue
IDC = Invasive ductal carcinoma
IDC (n) = Invasive ductal carcinoma necrosis
AT (DE) = Cancer adjacent breast tissue with ductal ectasia
An (FF): Cancer adjacent normal breast tissue (fibrofatty tissue and blood vessel) (fibro fatty tissue and blood vessel)
NA = Not available
Exp = expression
Loc = localization
C = cytoplasmic
N = nuclear
TNM = (T - Primary tumor, N - Regional lymph nodes, M - Distant metastasis)

Supplementary Table 3: Selected phosphopeptide deregulated by FRK on the kinome array

Signaling intermediate	Accession number	Peptide sequence	Phosphosite	Site implication	Effect of FRK
STAT3	P40763	DPGSAAPYLKTKFIC	Y705	Activating	Hypophosphorylation
STAT1	P42224	DGPKGTGYIKTELIS	Y701	Activating	Hypophosphorylation
JAK1	P23458	AIETDKEYYTVKDDR	Y1034/Y1035	Activating	Hypophosphorylation
JNK1	P45983	AGTSFMMTPYVVTRY	T183	Activating	Hypophosphorylation
CRK	P46108	GGPEPGPYAQPSVNT	Y221	Altered cell adhesion	Hyperphosphorylation
GRB10	Q13322	PELCGPGSPPVLTPG	S150	Activating	Hyperphosphorylation
GRB2	P62993	EECDQNWYKAELNGK	Y37	Increased cell growth	Hyperphosphorylation
AKT	P31749	RPHFPQFSYSASGTA	S473	Activating	Hypophosphorylation
MEK1	Q02750	VSGQLIDSMANSFVG	S217	Activating	Hypophosphorylation
P38	O15264	ADAEMTGYVVTRWYR	T180/Y182	Activation	Hypophosphorylation
